# The impact of C-tactile low-threshold mechanoreceptors on affective touch and social interactions in mice

**DOI:** 10.1126/sciadv.abo7566

**Published:** 2022-06-29

**Authors:** Damien Huzard, Miquel Martin, François Maingret, Jean Chemin, Freddy Jeanneteau, Pierre-François Mery, Pascal Fossat, Emmanuel Bourinet, Amaury François

**Affiliations:** 1 Institut de Génomique Fonctionnelle (IGF), Université de Montpellier, CNRS, INSERM, Montpellier, France.; 2 Institut des Maladies Neurodégénératives, Université de Bordeaux, CNRS, Bordeaux, France.

## Abstract

Affective touch is necessary for proper neurodevelopment and sociability. However, it remains unclear how the neurons innervating the skin detect affective and social behaviors. The C low-threshold mechanoreceptors (C-LTMRs), a specific population of somatosensory neurons in mice, appear particularly well suited, physiologically and anatomically, to perceive affective and social touch. However, their contribution to sociability has not been resolved yet. Our observations revealed that C-LTMR functional deficiency induced social isolation and reduced tactile interactions in adulthood. Conversely, transient increase in C-LTMR excitability in adults, using chemogenetics, was rewarding, promoted touch-seeking behaviors, and had prosocial influences on group dynamics. This work provides the first empirical evidence that specific peripheral inputs alone can drive complex social behaviors. It demonstrates the existence of a specialized neuronal circuit, originating in the skin, wired to promote interactions with other individuals.

## Teaser

A dedicated population of primary sensory neurons innervating the skin is tuned to promote sociability in mice.

## INTRODUCTION

The emotional value emerging from touch is important for decision making and motivation (*1*–*4*), especially in social animals, and dysregulation of this process may lead to debilitating psychiatric or neurologic conditions, including autism, anxiety, or depression (*5*–*8*). Nonetheless, the neural mechanisms underlying emotional tactile sensory processing relationship with social behavior are at the early stage of their understanding.

The skin is innervated by an array of functionally distinct populations of receptors contributing to touch. They can be distinguished by their response properties, activation threshold, conduction velocity (CV), and the type of end organ that they innervate (*9*).

One class of these receptors, the C-tactile (CT) fibers, is particularly well responsive to tactile stimulation categorized as “pleasant” and “affective” by human subjects (*10*, *11*). These neurons are unmyelinated low-threshold mechanoreceptors (LTMRs) and respond to nonnoxious touch with a predilection for slow-moving and low-force, stroking stimuli, such as gentle stroking and brushing (*11*–*13*). Activation of CTs in humans provides poor conscious spatial and qualitative information to the subjects, who nonetheless still carry a positive feeling related to the gentle brushing of their skin (*14*). These properties make them particularly well suited to link tactile information to social bonding (*15*, *16*).

In addition, it was found in laboratory animal models that pleasant touch contributes to sociability and related disorders. For example, playful and pleasant touch is rewarding and contributes to the social development of adolescent rats (*1*). In mice, two recent studies linked the disruption of LTMR functions with alterations of social behavior, typical of autism spectrum disorder (ASD) (*8*, *17*). Specifically, these studies used genetically modified mice recapitulating mutations found in human patients within the *Mecp2*, *Shank3B*, and *Fmr1* genes. Peripherally restricted conditional knockout (cKO) of these genes greatly altered the function of LTMRs, which induced multiple phenotypes associated with ASD, especially social deficits that are usually observed in constitutive KOs of these genes. However, LTMR sensory neurons are known to be a highly heterogeneous population, leaving open the question regarding the contribution of CT and pleasant touch to these social deficits.

Several groups genetically identified a population of primary sensory neurons in mice, named C-LTMRs, with similar functional properties than CTs. In rodents, C-LTMRs express specific set of genes allowing to differentiate them from other sensory neurons, such as tyrosine hydroxylase (TH), VGlut3, TAFA4, and Ca_v_3.2 (*18*–*23*). Study of the genes that may be used to genetically access to C-LTMRs unveiled their contribution to pain chronification in the context of neuropathic or inflammatory pathological pain. However, none of these studies considered the role of C-LTMRs in touch sensation within nonpathological conditions or their role in social behaviors.

In the present study, we explored the role of C-LTMRs in social interactions. We used two transgenic mouse models to decrease or facilitate C-LTMR excitability, combined with a tracking system automatically annotating social behaviors in social groups. Our investigation revealed the specific function of C-LTMRs in rodent interindividual relationships.

## RESULTS

### Impaired C-LTMR function in Ca_v_3.2^Nav1.8^cKO mice

In this study, we first examined the consequence of C-LTMR hypofunction on social behavior. For that purpose, we used a genetic mouse model in which the expression of the low-threshold calcium channel Ca_v_3.2 is conditionally knocked out in C-LTMRs, by crossing Ca_v_3.2^GFP-flox^KI mice with Nav1.8^cre^ mice, as previously described (*19*). Ca_v_3.2 is a proexcitatory voltage-gated calcium channel expressed in Aβ rapidly adapting LTMRs, D-Hair Aδ LTMRs, and C-LTMRs (*19*, *24*). The Ca_v_3.2^GFP-flox^KI mouse line has a green fluorescent protein (GFP) sequence fused in frame of the large extracellular loop of the channel first domain. This knock-in (KI) is targeted to *cacna1h* sixth exon and flanked by two LoxP sites to generate cKO when combined with mouse lines expressing the Cre recombinase. By using the Nav1.8^Cre^ mouse line combined with the Ca_v_3.2^GFP-flox^KI mouse model, Ca_v_3.2 expression should be specifically knocked out of C-LTMRs around E16 to E17, as soon as the Nav1.8 promoter starts to be active and drive Cre recombinase expression (*25*, *26*). This mouse model is named Ca_v_3.2^Nav1.8^cKO thereafter. To confirm that the Ca_v_3.2 channel is not expressed in C-LTMRs anymore, we performed immunofluorescence analysis of Ca_v_3.2^GFP-flox^KI and Ca_v_3.2^Nav1.8^cKO dorsal root ganglions (DRGs) at distinct postnatal stages (P0, P10, and P56). Since postnatal maturation of C-LTMR transcriptomic identity shows a dynamic expression of several fully differentiated C-LTMR markers (*27*), we used TH, the canonical marker of adult C-LTMRs at P56, in addition to GINIP and IB4 for adulthood, and earlier postnatal ages when TH is not expressed (*28*).

**Fig. 1.**
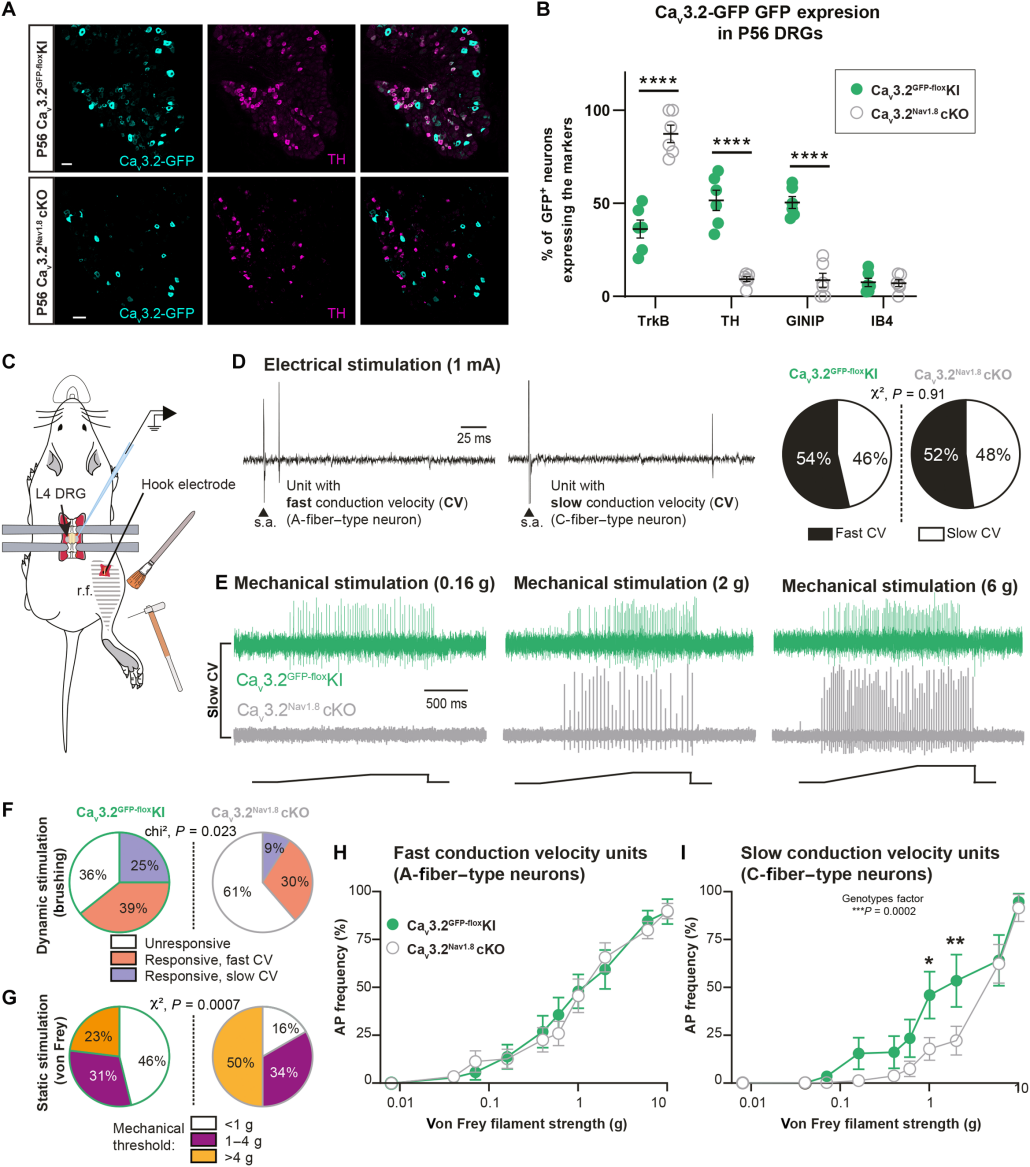
Ca_v_3.2 deletion in C-LTMRs altered their physiological responses to touch. (**A**) Expression of Ca_v_3.2-GFP (in cyan) in TH DRG neurons (in magenta) at P56 in Ca_v_3.2^GFP-flox^KI (top) and Ca_v_3.2^Nav1.8^cKO (bottom) mice. Scale bars, 50 μm. (**B**) Proportion of Ca_v_3.2-GFP^+^ DRG neurons expressing TrkB, TH, GINIP, or IB4 at P56 in Ca_v_3.2^Nav1.8^cKO (open gray circles) and Ca_v_3.2^GFP-flox^KI (green circles) mice. Two-way analysis of variance (ANOVA) with Sidak post hoc, ****P < 0.0001. *n* = 6 animals (three slides per animals). (**C**) Illustration of extracellular single-unit in vivo DRG recordings (r.f., receptive field). (**D**) Examples of single unit evoked by electrical stimulation (1 mA) of the sciatic nerve with fast CV (left) or slow CV (middle) (s.a., stimulation artifact). Right: Distribution of fast and slow CV units in Ca_v_3.2^GFP-flox^KI (fast CV, *n* = 15; slow CV, *n* = 13) and Ca_v_3.2^Nav1.8^cKO (fast CV, *n* = 23; slow CV, *n* = 21). (**E**) Examples of slowly conducting single unit evoked by von Frey filaments of different forces (0.16, 0.2, and 6 g) in Ca_v_3.2^GFP-flox^KI (green, upper traces) and Ca_v_3.2^Nav1.8^cKO (gray, lower traces). (**F**) Proportion of units responding to gentle brushing of the skin in Ca_v_3.2^Nav1.8^cKO and Ca_v_3.2^GFP-flox^KI mice. χ^2^ test, *P* = 0.023. (**G**) Proportion of units responding to static stimulation of the skin in Ca_v_3.2^Nav1.8^cKO and Ca_v_3.2^GFP-flox^KI mice. χ^2^ test, *P* = 0.007. (**H**) Action potential (AP) firing frequency in fast CV units in response to increasing mechanical stimulation in Ca_v_3.2^GFP-flox^KI (green circles; *n* = 14) and Ca_v_3.2^Nav1.8^cKO mice (open gray circles; *n* = 21). (**I**) AP firing frequency in slow CV units in response to increasing mechanical stimulation in Ca_v_3.2^Nav1.8^cKO (*n* = 13) compared to Ca_v_3.2^GFP-flox^KI (*n* = 18) mice. Two-way ANOVA (genotype factor: *P* = 0.0002), Sidak post hoc test. Ca_v_3.2^GFP-flox^KI versus Ca_v_3.2^Nav1.8^cKO; von Frey 1 g, *P = 0.0195 and von Frey 2 g, **P = 0.0081.

Our observation confirmed that at P56, in Ca_v_3.2^GFP-flox^KI control mice, 51.5% of all Ca_v_3.2-GFP^+^ neurons were TH^+^ (Fig. 1, A and B). We also confirmed that Ca_v_3.2 was expressed in 95.4% of all TH^+^ neurons at P56 (fig. S1D). In addition, we observed that 50.4% of all Ca_v_3.2-GFP^+^ neurons were also expressing GINIP, with almost no colabeling with IB4 (7.5% at P56; Fig. 1B and fig. S1, B, E, and F). Last, the GFP tagged to Ca_v_3.2 was observed in 94% of all TrkB^+^ neurons, a marker of Aβ rapidly adapting LTMRs and D-Hair Aδ LTMRs (fig. S1, A and C) (*29*). Ca_v_3.2-GFP^+^/TrkB^+^ neurons represented 36.1% of all Ca_v_3.2^+^ neurons at P56 (Fig. 1B and fig. S1C).

However, in DRGs from Ca_v_3.2^Nav1.8^cKO mice, we observed a markedly different distribution at P56. We detected almost no GFP in TH^+^ neurons (9% of all GFP^+^ neurons) or in GINIP^+^ neurons (2.8% of all GINIP^+^ neurons) (Fig. 1, A and B, and figs. S1B and S3, D to F). Moreover, Ca_v_3.2-GFP was mainly observed in TrkB^+^ neurons in the DRG of Ca_v_3.2^Nav1.8^cKO mice (87% of all GFP^+^ neurons; Fig. 1B and fig. S1, A and C).

To characterize when Ca_v_3.2 expression started to be affected in Cav3.2^Nav1.8^cKO mice, we performed a similar analysis at P0 and P10 in both Ca_v_3.2^GFP-flox^KI control mice and Ca_v_3.2^Nav1.8^cKO mice. It is worth noticing that TH is weakly expressed in DRG at P0 and P10 and is not an appropriate marker for C-LTMRs at these early postnatal time points. Therefore, to confirm the expression of Cav3.2 in C-LTMRs, we used a combination of GINIP and IB4, since C-LTMRs are the only peripheral neurons expressing GINIP without binding to IB4 throughout development (*27*, *28*). In Ca_v_3.2^GFP-flox^KI control mice, we observed that 49.9 and 45.7% of all Ca_v_3.2-GFP^+^ neurons were also expressing GINIP at P0 and P10, respectively, with almost no colabeling with IB4 (0% at P0 and 3.9% at P10). Conversely, Ca_v_3.2-GFP was observed in few GINIP^+^ neurons in P0 and P10 Ca_v_3.2^Nav1.8^cKO DRGs (16.1 and 9% of all GINIP^+^ neurons, respectively; figs. S1, E and F; S2, B and C; and S3, B and C). At P0, as at P10 and P56, TrkB^+^ neurons were the major DRG population expressing Ca_v_3.2 in Ca_v_3.2^Nav1.8^cKO mice (81.9, 70.7, and 87.3%, respectively; figs. S1, A and C, S2A, and S3A).

To confirm the functional expression of Ca_v_3.2 in DRG at P10 and infer on its potential role in C-LTMR postnatal development, we performed whole-cell patch clamp recordings in cultured DRGs from Ca_v_3.2^GFP-flox^KI mice at P10 and P56. At P56, in small-diameter (<20 μm) IB4^−^ and GFP^+^ neurons, we observed low voltage–activated (LVA) currents representing a large part of calcium voltage–activated currents (fig. S3D). More surprisingly, the amplitude of LVA current of small-diameter IB4^−^ and GFP^+^ neurons was much smaller at P10 in comparison to those observed at P56 (fig. S3, D and E). Thus, Ca_v_3.2 contribution to C-LTMR excitability is probably more important at adulthood than early in life.

To better assess the functional consequences of Ca_v_3.2 cKO in C-LTMRs, we investigated the physiological properties of LTMRs in Ca_v_3.2^GFP-flox^KI and Ca_v_3.2^Nav1.8^cKO mice using in vivo single-unit recordings in anesthetized animals (Fig. 1C). We inserted an electrode in L4 DRG and differentiated units with fast CV typical of Aβ and Aδ fiber, and units with slow CV typical of C-fibers (Fig. 1D) that responded to dynamic (gentle brushing) and punctate (von Frey filaments) mechanical stimulations (Fig. 1E). The delay of responses was measured as the delay between the stimulation artifact and the action potential recorded. As illustrated in Fig. 1F, the occurrence of single-unit responses to skin brushing in DRGs was markedly reduced in Ca_v_3.2^Nav1.8^cKO mice (Ca_v_3.2^GFP-flox^KI mice: brush responsive units, *n* = 18 of 28 = 64%; brush unresponsive units, *n* = 10 of 28 = 36%; Ca_v_3.2^Nav1.8^cKO mice: brush responsive units, *n* = 17 of 44 = 39%; brush unresponsive units, *n* = 27 of 44 = 61%). Further analyses confirmed that this effect is due to a reduction in the number of slowly conductive C-fibers responding to skin brushing in Ca_v_3.2^Nav1.8^cKO compared to Ca_v_3.2^GFP-flox^KI (slow CV units of Ca_v_3.2^Nav1.8^cKO, *n* = 4 of 44 = 9%; Ca_v_3.2^GFP-flox^KI mice, *n* = 7 of 27 = 25%; χ^2^ = 7.532; *P* = 0.023 Fig. 1F). Furthermore, a proportion of slow CV units with a response threshold below 1 g was decreased in Ca_v_3.2^Nav1.8^cKO than in Ca_v_3.2^GFP-flox^KI mice and half of these units had a threshold above 4 g in Ca_v_3.2^Nav1.8^cKO mice [threshold < 1 g (Ca_v_3.2^GFP-flox^KI, *n* = 6 of 13 = 46%; Ca_v_3.2^Nav1.8^cKO, *n* = 3 of 18 = 16%); 1 g < threshold < 4 g (Ca_v_3.2^GFP-flox^KI, *n* = 4 of 13 = 31%; Ca_v_3.2^Nav1.8^cKO, *n* = 6 of 18 = 34%); threshold > 4 g (Ca_v_3.2^GFP-flox^KI, *n* = 3 of 13 = 23%; Ca_v_3.2^Nav1.8^cKO, *n* = 9 of 18 = 50%; χ^2^ = 14.5; *P* = 0.0007; Fig. 1, E and G]. In addition, the firing responses of Ca_v_3.2^Nav1.8^cKO neurons were blunted (*P* = 0.0002), especially to von Frey filaments of 1 and 2 g (Fig. 1I). Fast CV A-LTMR sensitivity and responses were not affected (Fig. 1H). These differences between the two genotypes point toward a hypofunction of C-LTMRs in adult Ca_v_3.2^Nav1.8^cKO mice. The mutation did not affect the response latency to electrical stimulation of fast [Ca_v_3.2^GFP-flox^KI: 94 ± 12 ms (*n* = 13) versus Ca_v_3.2^Nav1.8^cKO: 93 ± 15 ms (*n* = 17)] and slow [Ca_v_3.2^GFP-flox^KI 8.2 ± 1.4 ms (*n* = 14) versus Ca_v_3.2^Nav1.8^cKO: 8.7 ± 1.2 ms (*n* = 16)] conductive units. Together, our results indicate that Ca_v_3.2 deletion in C-LTMRs impaired their function at adulthood, especially for the detection of slow dynamic simulation and gentle punctate stimulation without affecting myelinated LTMRs.

### Social behaviors are impaired in Ca_v_3.2^Nav1.8^cKO

**Fig. 2.**
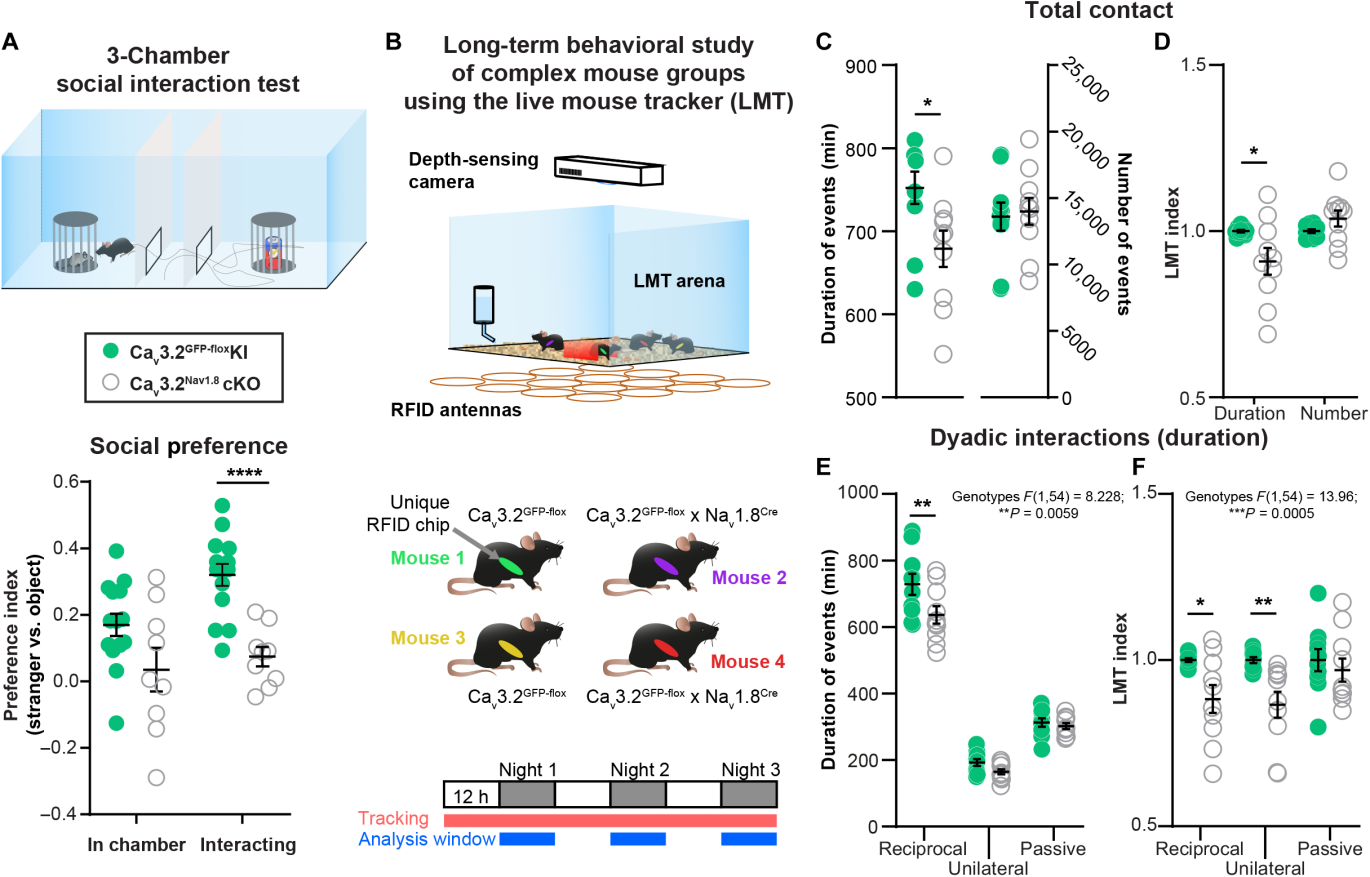
C-LTMR impairment via Ca_v_3.2 deletion reduces social behaviors. (**A**) In the three-chamber social preference test, Ca_v_3.2^Nav1.8^cKO mice had a decreased preference toward a stranger mouse in comparison to control Ca_v_3.2^GFP-flox^KI littermates. Unpaired *t* test *P* < 0.001: Ca_v_3.2^GFP-flox^KI, *n* = 14 (light green circles); Ca_v_3.2^Nav1.8^cKO, *n* = 9 (open gray circles). (**B**) Live Mouse Tracker (LMT) schematic description. Each group of mice includes two Ca_v_3.2^GFP-flox^KI and two Ca_v_3.2^Nav1.8^cKO mice. The experiment was performed over a 3-day period, and each behavior was analyzed from the dark (“active”) phase of the light cycle. (**C**) Duration and the number of all contacts automatically extracted by the LMT of Ca_v_3.2^GFP-flox^KI (light green circles; *n* = 10) and Ca_v_3.2^Nav1.8^cKO (open gray circles; *n* = 10). Sum of all the events for the three nights. Unpaired *t* test corrected for multiple comparisons using the Holm-Sidak method. Adjusted *P* value: *P = 0.01969. (**D**) LMT indexes of Ca_v_3.2^GFP-flox^KI (light green circles; *n* = 10) and Ca_v_3.2^Nav1.8^cKO (open gray circles; *n* = 10) for the duration and the number of all contacts. Sum of all the events for the three nights. Unpaired *t* test corrected for multiple comparisons using the Holm-Sidak method. Adjusted *P* value: *P = 0.0206. (**E**) Duration of dyadic interactions occurring in the LMT: reciprocal, unilateral, and passive interactions of Ca_v_3.2^GFP-flox^KI (light green circles; *n* = 10) and Ca_v_3.2^Nav1.8^cKO (open gray circles; *n* = 10; **P = 0.0096). (**F**) LMT indexes for dyadic interactions computed for Ca_v_3.2^GFP-flox^KI (light green circles; *n* = 10) and Ca_v_3.2^Nav1.8^cKO (open gray circles; *n* = 10) mice. The duration of events for the three dark phases was summed for statistical analysis. Two-way ANOVA with Sidak post hoc test *P = 0.028 and ****P = 0.0096. ANOVA results are indicated below the panel title, and full results are presented in table S1.

To evaluate the consequences of C-LTMR deficiency on social preference behavior, we used the three-chamber social preference paradigm. Male Ca_v_3.2^Nav1.8^cKO had a lower social preference index compared to control Ca_v_3.2^GFP-flox^KI littermates, illustrating a diminution of social interest toward an unfamiliar mouse in comparison with an unanimated object (Fig. 2A and fig. S4A). To further investigate the precise quality of the social and tactile interactions that are impaired in this model, we used a recently developed paradigm in which mice could interact freely with each other. The Live Mouse Tracker (LMT) is based on a machine learning analysis framework and was designed by de Chaumont and colleagues (*30*) for that specific purpose. This system allows the tracking and automatic annotation of mice behaviors and social interactions in their environment for multiple days (*30*). Using the LMT, we analyzed the behavior of five groups of four male mice (each group composed of two controls Ca_v_3.2^GFP-flox^KI and two C-LTMR–impaired Ca_v_3.2^Nav1.8^cKO, all littermates) for three consecutive nights (Fig. 2B). For every time frame, the LMT detects head, tail, ears, eyes, and nose position, producing a geometrical mask for each mouse. These data enable the computation of different behavioral events based on mice geometries, body movements, and localization compared to other mice. Overall, we categorized 28 events into seven categories: body configuration, isolated behavior, position in contact, type of contact, social configuration, social approach, and social escape. These events were analyzed separately for both number of occurrences and their duration for each individual (table S1). As previously published, the value of each behavioral trait for a mouse was normalized by the mean value of that trait from the two control mice of the same cage. This normalization (called “LMT index” thereafter) was allowed to control for both individual variability and the “cage effect” naturally occurring in each set of four mice.

The LMT indexes from Ca_v_3.2^GFP-flox^KI and Ca_v_3.2^Nav1.8^cKO mice indicated for the social events that Ca_v_3.2^Nav1.8^cKO mice, with impaired C-LTMRs, spent less time in contact (9% decrease in LMT index) than the control mice (Fig. 2, C and D, and table S1). Moreover, Ca_v_3.2^Nav1.8^cKO mice spent more time isolated than the controls (increase of 29.5%, average of all isolated behaviors; fig. S4D and table S1), without any noticeable differences in locomotor activity (fig. S4B) or exploratory behavior [stretch attending posture (SAP); fig. S4C and table S1]. In addition, the duration of reciprocal (associated with nose-to-nose or side-by-side contacts) and unilateral social interactions (associated with ano-genital contacts) were reduced by 11 and 13%, respectively (Fig. 2, E and F, fig. S4F, and table S1), in C-LTMR–impaired animals. However, passive social interaction (associated in our experiment with receiving ano-genital contacts) was not altered (Fig. 2, E and F, and table S1). Furthermore, no differences were observed in the social approaches or social configuration behavior categories (fig. S4, G and H, and table S1). Together, the results from the social preference test and the LMT revealed a deficit in sociability of Ca_v_3.2^Nav1.8^cKO mice compared to their control littermates.

### A novel viral strategy to specifically target C-LTMRs in mice

**Fig. 3.**
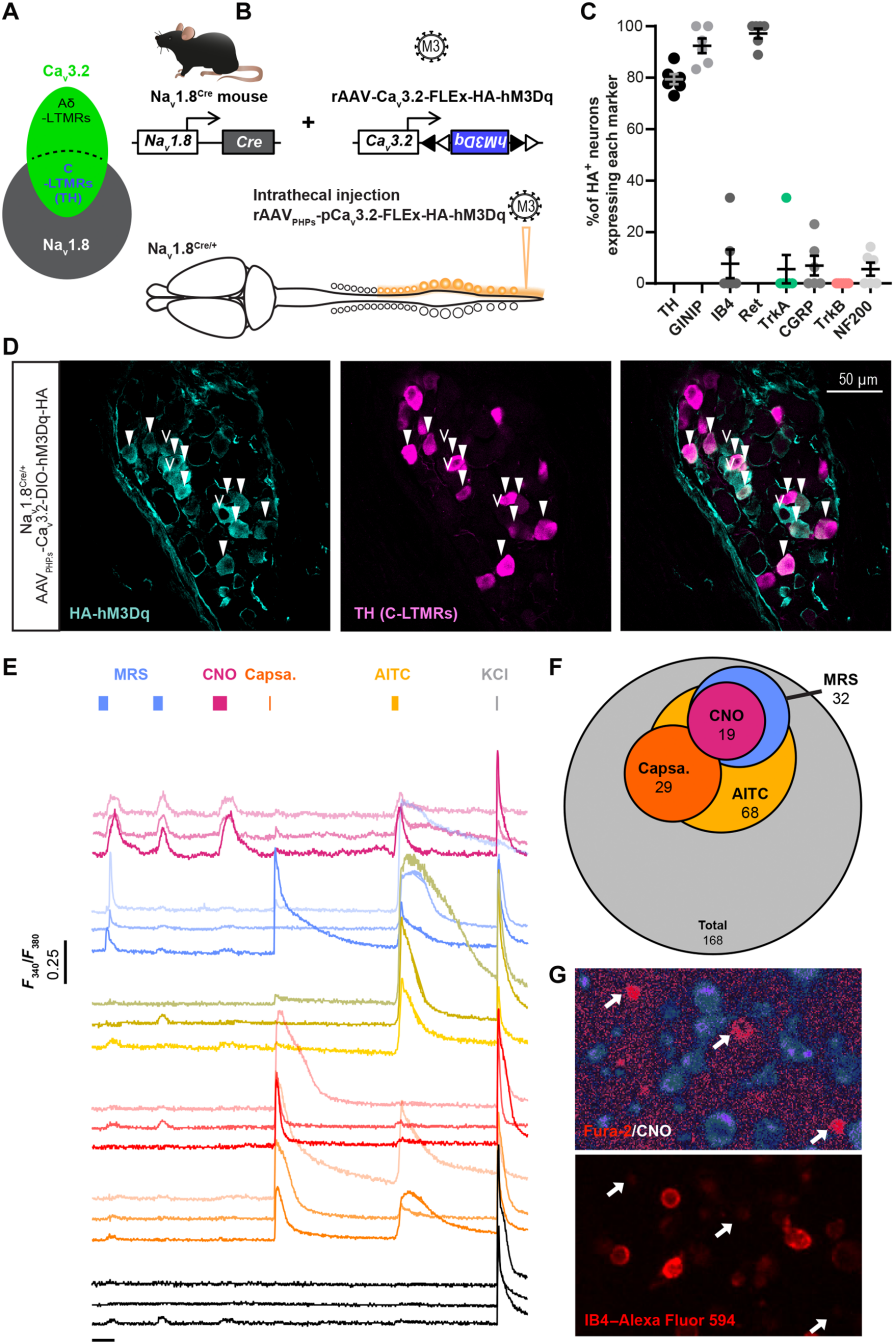
Viral expression of DREADD receptors HA-hM3Dq in C-LTMRs. (**A**) Diagrams representing primary sensory neurons expressing Nav1.8 and Ca_v_3.2 and the population defined by the expression of both ion channels. (**B**) Strategy to express HA-hM3Dq in C-LTMRs using intrathecal injection of AAV_PHPs_ serotypes in Nav1.8^Cre^ mice. (**C**) Bar graph of the percentage of hemagglutinin (HA)–positive (HA^+^) neurons expressing each of the DRG population markers indicated in the *x* axis. Each dot represents one mouse (three sections counted per mice), *n* = 6 mice. (**D**) Representative images of an immunofluorescence of TH (C-LTMRs marker, magenta) and HA tag (HA-hM3Dq, cyan) in thoracic DRG (T13) of a Nav1.8^Cre/+^ mouse injected with AAV_PHPs_-pCa_v_3.2-FLEx-HA-hM3Dq. Filled white arrowheads indicate examples of neurons positive for HA and TH. Empty arrowheads indicate examples of neurons positive for HA only. (**E**) Fura-2 representative individual calcium influx in cultured DRG neurons from Nav1.8^Cre/+^ mice infected with AAV_PHPs_-pCa_v_3.2-FLEx-HA-hM3Dq, following bath perfusion of MRS2365 (MRS; 200 nM, blue), clozapine *N*-oxide (CNO) (30 μM, purple), capsaicin (Capsa., 500 nM, orange), AITC (200 μM, yellow), or KCl (40 mM, gray). (**F**) DRG neurons from Nav1.8^Cre/+^ mice infected with AAV_PHPs_-pCa_v_3.2-FLEx-HA-hM3Dq responding to CNO also respond to MRS and AITC. Venn diagram for the number of neurons responding to the different chemical compounds. CNO responders (purple, *n* = 19), MRS responders (blue, *n* = 32), AITC responders (yellow, *n* = 68), and capsaicin responders (orange, *n* = 29). In total, *N* = 168 neurons were recorded from three animals. (**G**) CNO responding neurons do not bind to IB4. Representative images of ratiometric calcium imaging on cultured DRG from a Nav1.8^Cre^ mice injected with AAV_PHPs_-pCa_v_3.2-FLEx-HA-hM3Dq. Top: Ratiometric Fura-2 after CNO bath perfusion. Bottom: Live IB4–Alexa Fluor 594 staining of the same field of view. White arrows indicate CNO-responsive DRG neurons.

Next, we deepened our investigations on the role of C-LTMR in sociability by designing a chemogenetic strategy to selectively excite C-LTMRs remotely in adult mice independently of Ca_v_3.2 protein function and without any postnatal functional perturbation of C-LTMR. Our strategy to target C-LTMRs in adult mice, illustrated in Fig. 3 (A and B), consisted of expressing our gene of interest (hM3Dq) under the control of the mini-Ca_v_3.2 promoter in a Cre-dependent manner. C-LTMRs can be defined by the expression of both the sodium channel Nav1.8 and the calcium channel Ca_v_3.2 (*19*, *31*). We previously engineered adeno-associated viruses (AAVs) with a mini-Ca_v_3.2 promoter sequence and validated its faithful expression in Ca_v_3.2-positive neurons, within the dorsal horn of the spinal cord (*32*). Here, we used an AAV-PHP-S serotype that has a high tropism for peripheral sensory neurons (*33*), which we delivered into Nav1.8^Cre^ heterozygote mice. This intersectional strategy combining the expression pattern of Ca_v_3.2 and Nav1.8 aimed at restricting the expression of the viral payload into C-LTMRs. To achieve C-LTMR chemogenetic stimulation with this strategy, we inserted the hemagglutinin (HA)–tagged hM3Dq excitatory DREADD cassette within the pAAV vector.

To validate the Cre dependency and functionality of our construct, we first transfected human embryonic kidney (HEK) 293T cells with the pAAV vector (pAAV-pCa_v_3.2-FLEx-HA-hM3Dq). As the Ca_v_3.2 promoter activity is highly enhanced by the EGR1 transcription factor (*34*), we coexpressed the murine EGR1 cDNA and added or not the Cre recombinase (Cre-GFP fusion). We analyzed the DREADD functionality using calcium fluorimetry in Cre-GFP–positive HEK cells loaded with Fura-2. In a representative field, out of 102 HEK cells, 28 were GFP positive, and from those, 17 responded to the hM3Dq pharmacological actuator clozapine *N*-oxide (CNO) (fig. S6, A and B). No responses to CNO were observed in non-GFP HEK cells.

Then, we packaged the viral particles with this construct using an AAV-PHP-S capsid. Although AAV PHP-S can be delivered systemically to access sensory neurons, we injected the virus intrathecally with minimal invasiveness and a sole access to DRGs and no other sensory neurons such as those of the vagal ganglia (Fig. 3B) (*33*). Consistent with an efficient C-LTMR targeting strategy, intrathecal injection of this viral construct leads to the expression of the excitatory DREADD receptor HA-hM3Dq in C-LTMRs labeled by TH, 8 weeks after injection (Fig. 3D). Overall, 79.4% of HA-hM3Dq cells were also positive to TH, and 92% of these neurons were also GINIP^+^ but not IB4, indicative of their C-LTMR phenotype (*n* = 6 mice; with three sections per mouse) (*28*). HA^+^ neurons were all c-ret^+^ neurons (97%), but almost none were IB4^+^ (7.6%). We also observed in rare occasion a colabeling of HA with CGRP or TrkA (6.9 and 5.5%, respectively), presumably C-LTMRs innervating the colon or the bladder (*35*), as well as in NF200 neurons (5.5%), which were not TrkB^+^ neurons (*n* = 6 mice; with three sections per mouse) (Fig. 3C and fig. S5). Inversely, HA-hM3Dq is present in 31.9% of TH^+^ dorsal root ganglia and 16.6 ± 1.2% GINIP^+^ neurons (fig. S5H). In addition, no observation was made of any specific HA tag immunostaining in the dorsal horn of the spinal cord (fig. S6D).

To further confirm that hM3Dq was selectively expressed in C-LTMRs according to the defined strategy and kept its proexcitatory nature, we evaluated the effect of CNO directly on DRG neurons. We dissected out lumbar and sacral DRGs from animals injected intrathecally with rAAV_PHPs_-pCa_v_3.2-FLEx-HA-hM3Dq viruses 6 to 8 weeks before. Then, we dissociated DRG neurons and maintained them in short-term culture (12 hours). Last, we loaded them with the Fura-2 ratiometric calcium indicator and labeled with red dye–conjugated IB4 to assess the pharmacological and functional properties of large population of neurons at the same time. Application of CNO (30 μM) induced an intracellular calcium increase in neurons also responding to the TRPA1 agonist, allyl isothiocyanate (AITC; 200 μM), and to the P2Y1R agonist, MRS2365 (MRS; 200 nM) (Fig. 3, E and F). From all the neurons responding to CNO, the large majority responded to AITC and MRS (*15*), three neurons also responded to capsaicin on top of AITC and MRS, and only one was not responding to anything else. Among the MRS responders, 56.2% were also responding to CNO, while among AITC responders only 26.4% were CNO responders (Fig. 3, E and F, and fig. S6C). In mouse DRGs, TRPA1 is weakly expressed in C-LTMRs and P2YR1 receptor is only expressed in C-LTMRs and TrkB^+^ Aδ-LTMRs (*21*, *23*). As TRPA1 is not expressed in TrkB neurons, responses to both MRS and AITC can only be observed in C-LTMRs. Moreover, none of the CNO-responsive neurons were labeled by IB4 (Fig. 3G), in agreement with the lack of reactivity of lectin in mouse TH-positive C-LTMR neurons (*36*). Together, our morphological and functional data provide supporting evidence toward a specific expression of HA-hM3Dq in a large population of C-LTMRs (10.7% of all DRG neurons recorded, *N* = 3 mice). Accordingly, we used this experimental approach in vivo to investigate the impact of C-LTMR stimulation in social behaviors.

### Effect of exogenous C-LTMR activation on somatosensory perception

First, we assessed the consequences of C-LTMR exogenous activation on somatosensory perception. Eight weeks after intrathecal injections of rAAV_PHPs_-pCa_v_3.2-FLEx-HA-hM3Dq (named C-LTMRs^hM3Dq^ mice) or rAAV_PHPs_-CAG-mCherry (control mice) in Nav1.8^cre^ mice, we administrated CNO intraperitoneally (1 mg/kg) to all animals. At 30 to 45 min after injection, we observed a decrease in the paw withdrawal reflex frequency responses to low-force von Frey stimulation (filament of 0.07 g) in C-LTMRs^hM3Dq^ mice compared to control mice, probably due to the fact that they are already activated by CNO. However, no difference was observed for higher forces (0.6 and 2 g) or for brushing (fig. S7A). Similarly, CNO injection did not trigger any alteration of motor activity or spontaneous nocifensive behaviors, such as paw shaking, guarding, grooming, licking, or jumping.

**Fig. 4.**
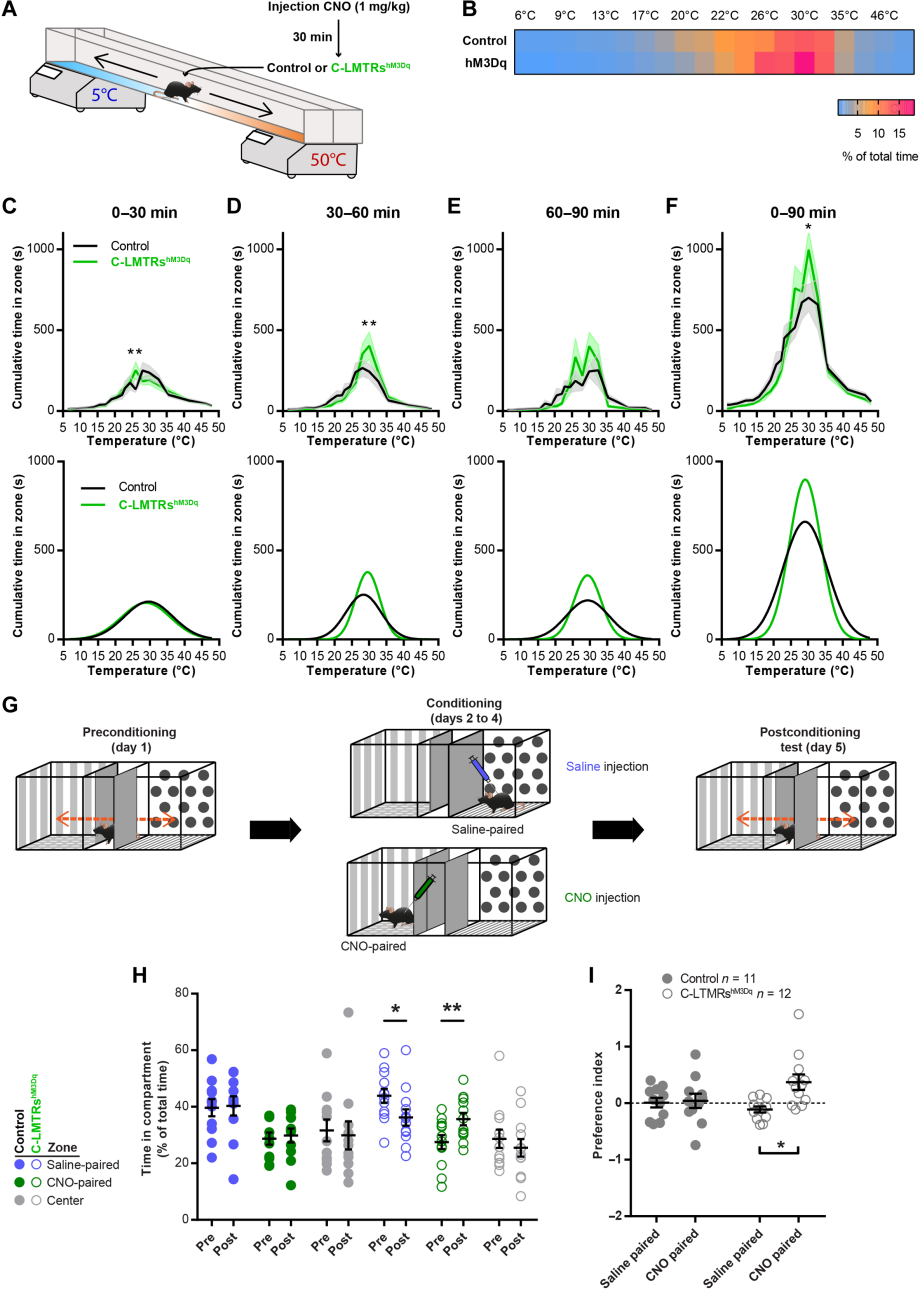
C-LTMR exogenous activation increases thermal preference and induced conditioned place preference. (**A**) Thermal gradient protocol to assess thermotaxis of control (Nav1.8^Cre^; AAV_PHPs_-CAG-mCherry; in black) and C-LTMRs^hM3Dq^ (Nav1.8^Cre^; AAV_PHPs_-pCa_v_3.2-FLEx-HA-hM3Dq; in green) mice. (**B**) Heatmap of the zone occupancy (in % or total time) in the thermal gradient for 90 min after CNO injection. (**C**) Top: Cumulative time in the thermal gradient zones during the first 30 min after CNO injection. Bottom: Predictive curves based on a Gaussian fit of the results. (**D**) Top: Cumulative time in the thermal gradient zones between 30 and 60 min after CNO injection. Bottom: Predictive curves based on a Gaussian fit of the results. (**E**) Top: Cumulative time in the thermal gradient zones between 60 and 90 min after CNO injection. Bottom: Predictive curves based on a Gaussian fit of the results. (**F**) Top: Cumulative time in the thermal gradient zones for the entire 90 min after CNO injection. Bottom: Predictive curves based on a Gaussian fit of the results. Statistics performed: two-way ANOVA, Bonferroni post hoc test, **P = 0.0037 (C); **P = 0.0019 (D); *P = 0.0112 (F); control, *n* = 11 and C-LTMRs^hM3Dq^, *n* = 12. (**G**) Conditioned place preference protocol. (**H**) Time spent in compartments before (pre-) and after (post-) 3 days of conditioning. For each condition: two-way repeated-measures (RM) ANOVA, Sidak post hoc test [**P = 0.0098; *P = 0.0143; control (*n* = 11) and C-LTMRs^hM3Dq^ (*n* = 12)]. (**I**) C-LTMRs^hM3Dq^ preferred the compartment in which they received CNO, while no preference was observed in control mice. Conditioned place preference index [(time post − time pre)/time pre], two-way RM ANOVA, Bonferroni post hoc test (*P = 0.0141).

As C-LTMRs have been implicated into temperature perception (*18*, *19*, *37*), we probed thermal sensitivity of control and C-LTMRs^hM3Dq^ mice in the thermal gradient test. Thirty minutes after CNO injection, mice were placed into a corridor (1.5 m long) with the floor regulated at 5° or 50°C at the extremities, creating a gradient of temperature. Once placed in the corridor, mice were allowed to explore the thermal gradient to reveal their thermotaxis behavior. The exploration was tracked during 90 min, and the animal’s position was annotated according to the temperature zones they visited (Fig. 4A). When compared to control animals, CNO-treated C-LTMRs^hM3Dq^ mice settled more quickly at the comfort temperature of 30°C (Fig. 4, C and D) and overall spent more time at this temperature (Fig. 4, B and F). There was no difference in locomotion (fig. S7, B and C), and both groups had the same preference in temperature (fig. S7D). Furthermore, no difference was observed when the animals were injected with saline (fig. S7, E to H). This result suggests that exogenous activation of C-LTMRs could reinforce preferences for an optimal somatosensory stimulus.

### Positive valent information is associated with C-LTMR stimulation

As the activation of C-LTMRs may results in a positive feeling, we investigated whether C-LTMR activation could be rewarding on its own by using the conditioned place preference (CPP) paradigm. Following 1 day of habituation to the CPP arena, control and C-LTMRs^hM3Dq^ mice were conditioned by receiving saline injection in the compartment they preferred during habituation and CNO injection (1 mg/kg diluted in saline) in the other compartment for 3 consecutive days (Fig. 4G). On the last day, mice were free to explore the entire arena and their position was video-tracked. While control animals did not develop a preference for a specific side, C-LTMRs^hM3Dq^ mice showed a marked preference for the compartment in which they received CNO injections (increase of 32%; Fig. 4, H and I). Overall, these two experiments suggest that the activation of C-LTMRs was perceived as rewarding and can increase the rewarding value of other sensory modalities.

### C-LTMR stimulation induces touch-seeking and prosocial behaviors

**Fig. 5.**
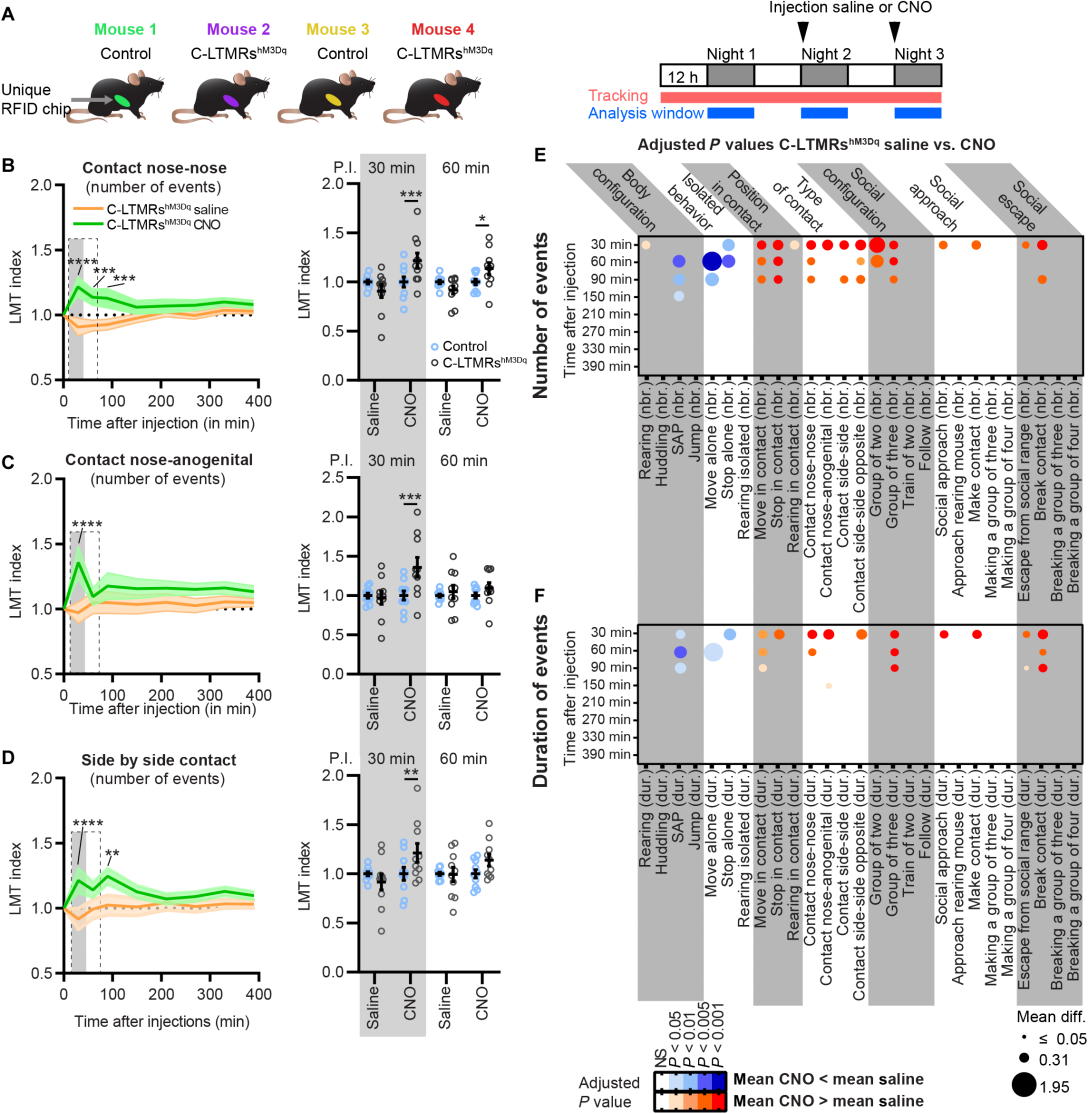
C-LTMR exogenous activation transiently increases contact between animals and reduces isolated behavior. (**A**) Left: Cage composition of each group analyzed in the LMT. Right: Injections, tracking, and analysis protocol. (**B** to **D**) Left: Time course of the LMT index obtained at 30, 60, 90, 150, 210, 270, 330, and 390 min after saline injection (orange trace) or after CNO injection (green trace) for the cumulative number of events for the nose-to-nose contacts (B), nose-to-anogenital contacts (C), and side-by-side contacts (D). Two-way RM ANOVA, LMT index C-LTMRs^hM3Dq^ CNO versus saline. Sidak post hoc test, *P < 0.05; **P < 0.01; ***P < 0.005; ****P < 0.0001. Right: LMT index obtained for the three types of contacts at 30 and 60 min post-injections (P.I.) in control (open blue circles) and C-LTMRs^hM3Dq^ (open black circles) mice. Two-way RM ANOVA, LMT index C-LTMRs^hM3Dq^ saline versus LMT index control saline and LMT index C-LTMRs^hM3Dq^ CNO versus LMT index control CNO. Sidak post hoc test, *P < 0.05; **P < 0.01; ***P < 0.005; ****P < 0.0001. Each dot represents one mouse. (**E** and **F**) Representation of the adjusted *P* value and the mean difference obtained during the time course by comparing the LMT indexes from C-LTMRs^hM3Dq^ after saline versus CNO injections. Two-way RM ANOVAs performed on each time course for every number (E) and duration (F) of events for each behavioral trait automatically annotated by the LMT. Sidak post hoc tests. The mean differences were indicated by the size of the dots, and the *P* values were color-coded depending on the level of significance and the polarity of the mean difference used for comparison: mean (CNO) > mean (saline) (shade of red) or mean (CNO) < mean (saline) (shade of blue), *n* = 10. All *P* values, confidence intervals, and mean differences are indicated in table S2.

Taking into consideration the intrinsic emotional value conveyed by C-LTMR activation, we finally investigated whether the activation of C-LTMRs can affect social behaviors and social group organization. The LMT system was again used to analyze the behavior of five groups of four mice independently during three nights. Each group was composed of two Nav1.8^cre^ mice injected with rAAV_PHPs_-CAG-mCherry (control mice) and two Nav1.8^cre^ mice injected with rAAV_PHPs_-pCa_v_3.2-FLEx-HA-hM3Dq (C-LTMRs^hM3Dq^ mice), all male littermates. The first 24 hours were used as a habituation phase, and just before the second dark cycle, we injected either saline or CNO (intraperitoneally, 1 mg/kg diluted in saline) to all mice (control and C-LTMRs^hM3Dq^). At the beginning of the third dark cycle, groups that received a saline solution for the second dark cycle received CNO and vice versa (Fig. 5A). The LMT index was calculated from cumulated value for both saline and CNO injection during the first half of the dark cycle, every 30 min between 0 and 150 min and every hour until 6.5 hours (390 min) after injection (Fig. 5, B to F). In comparison to saline injections, activation of C-LTMRs with CNO increased the amount of nose-to-nose, nose-to-anogenital, and side-by-side contacts (at 30-, 60-, and 90-min time points) (Fig. 5, B to D), Overall, CNO, but not saline injection, significantly reduced the number of isolated events, while it increased interindividual events in C-LTMRs^hM3Dq^ mice but not in controls 1 hour after injection (C-LTMRs^hM3Dq^ CNO injection versus LMT index C-LTMRs^hM3Dq^ saline; move alone: −122%, 60 min after CNO injection; stop alone: −50%, 60 min after CNO injection; move in contact: +24%, 60 min after CNO injection; stop in contact: +35%, 60 min after CNO injection; Fig. 5, E and F; fig. S8, B to D; and table S2).

We also observed that C-LTMRs^hM3Dq^ mice were engaging more and, for longer periods, in all the different types of contacts following the injection of CNO (Fig. 5, B to D). Specifically, we observed that behavioral traits associated with active social interaction were transiently altered, lasting for up to 30 to 90 min (number of contact nose-anogenital: +38%, 30 min after CNO injection; number of contact nose-nose: +21%, 60 min after CNO injection; number of contact side-by-side: +22%, 90 min after CNO injection; and number of contact side-by-side opposite: +26%, 90 min after CNO injection; Fig. 5, B to F; fig. S8, E to G; and table S2) in accordance with known CNO clearance time (*38*).

In conclusion, the activation of C-LTMRs transiently increased all kinds of social interactions between animals to the expense of isolated behavior for up to 90 min following CNO injections, including behaviors related to skin-to-skin contacts and social exploration. The impact of such behavioral alteration appears to impact group dynamics for similar periods, especially the groups-of-two and groups-of-three mice. All behavioral traits in C-LTMRs^hM3Dq^ mice came back to baseline 2.5 hours after injection.

**Fig. 6.**
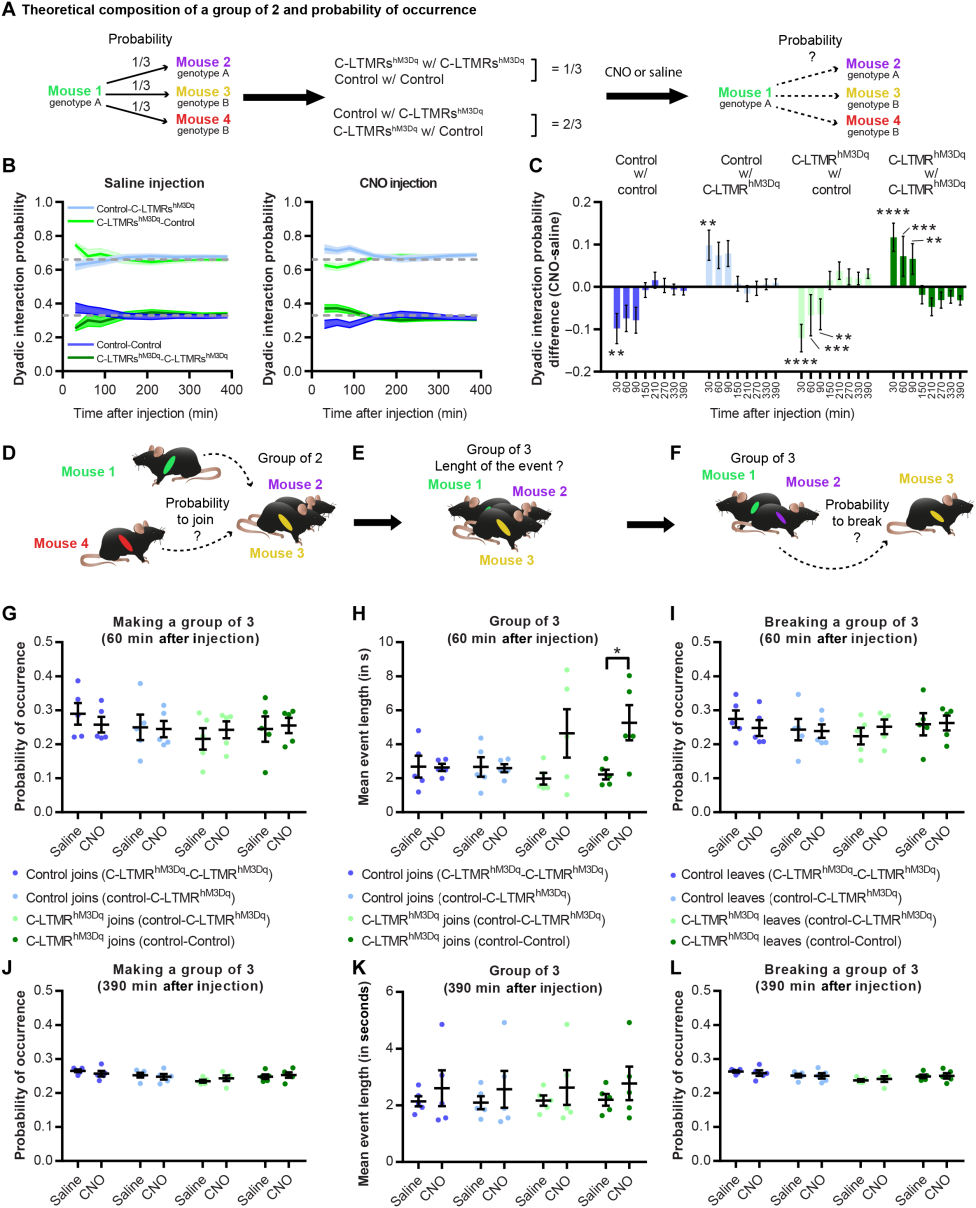
C-LTMR exogenous activation shakes group dynamics and interindividual interaction probabilities. (**A**) Theoretical composition of a group of two based on its probability of occurrence. (**B**) Dyadic interaction probability for all four possibilities at 30, 60, 90, 150, 210, 270, 330, and 390 min after saline (left) and CNO (right) injection. (**C**) Dyadic interaction probability difference between CNO and saline injections at 30, 60, 90, 150, 210, 270, 330, and 390 min after injection. Asterisks referred to the comparison between difference at baseline (T-1 hour before injection) and the difference for each time point for each group. Two-way RM ANOVA. Sidak post hoc test, **P < 0.01, ***P < 0.001 and ****P < 0.0001. (**D**) Probability of occurrence for a mouse of any given genotype to create a group of three of all possible compositions at 60 min (**G**) or 390 min (**J**) after saline or CNO injection. (**E**) Length of the group of three events created by a given mouse depending on the total composition of the group at 60 min (**H**) or 390 min (**K**) after saline or CNO injection. (**F**) Probability of occurrence for a mouse of any given genotype to break a group of three of all possible composition at 60 min (**I**) or 390 min (**L**) after saline or CNO injection. Paired *t* test, *P* = 0.0486.

In addition, we analyzed the relationship between each mouse and the group dynamics. First, we focused on groups of two mice and, in our condition, one mouse of a given genotype had a probability of ^1^/_3_ to interact with a mouse from the same genotype and ^2^/_3_ to interact with a mouse from the other genotype (Fig. 6A). Following CNO, but not saline, injection, C-LTMRs^hM3Dq^ mice interacted more with each other than expected by chance level (+11.7 ± 0.2% compared to saline), while control mice interacted less with each other (−9.8 ± 0.2% compared to saline) (Fig. 6, B and C). In addition, control mice had a higher probability of making contact with C-LTMRs^hM3Dq^ mice than chance level (+9.7 ± 0.3% compared to saline). This effect was visible for 30 and 90 min following CNO injection, depending on the dyad formed. These observations were valid only when all the types of dyadic contacts were analyzed together but not when behaviors related to social exploration were analyzed individually, in which case only C-LTMRs^hm3Dq^ mice had a higher probability of interacting with each other (fig. S9, A to D). However, the mean duration of time spent in groups of two was similar between C-LTMRs^hm3Dq^ and control mice. Next, we investigated the dynamic of groups of three mice, 60 min after injection (Fig. 6, G to I) and 390 min after injection (Fig. 6, J to L). While looking at the combination of mouse making or breaking groups of three, we did not observe any differences following CNO or saline injections (Fig. 6, D, F, G, I, J, and L), and this was the case at all time points. However, it appears that the groups of three mice formed by a C-LTMRs^hM3Dq^ mouse lasted longer than those created by a control mouse, especially if the C-LTMRs^hM3Dq^ mouse joined a mixed group of two (one C-LTMRs^hM3Dq^ mouse and one control; Fig. 6, E and H). This effect was particularly notable 1 hour after CNO injection but decreased after 90 min and was completely abolished after 390 min (Fig. 6K).

## DISCUSSION

Understanding how the sense of touch may shape social interactions, while keeping a level of ethological validity, is particularly challenging in laboratory conditions. In this study, we combined a unique genetic strategy with a tracking technology to characterize the contribution of C-LTMRs to affective and social touch in mice. By reducing the mechanical sensitivity of this specific population of primary sensory neurons, which were defined both physiologically and genetically, we observed in adults a reduction of social contacts with other mice, leading to an increase in isolated behaviors. Conversely, remote activation of C-LTMR with a chemogenetic approach in adults led to an increase of social interactions and a reduction of isolated behavior and changed groups’ social dynamics. We present evidence that mouse C-LTMRs may be one of the main contributors to social touch and a potential way to improve social deficits.

### Ca_v_3.2 as a key component of C-LTMR excitability

We and others documented the impact of Ca_v_3.2 in helping adult LTMR neurons to fire in burst by lowering action potential threshold and by generating after depolarization potentials (*19*, *24*, *39*, *40*). Accordingly, in the Ca_v_3.2^Nav1.8^cKO mouse line, C-LTMR remained mechanosensitive after Ca_v_3.2 cKO but responded with higher threshold to static mechanical stimuli as the result of dampened excitability [Fig. 1, F, G, and I, and (*19*)]. It seems that Ca_v_3.2 cKO in C-LTMRS markedly reduced the ability of these neurons to respond to dynamic mechanical stimulation. C-LTMR inhibition using Ca_v_3.2^Nav1.8^cKO mice is a cKO in which the expression of the proexcitatory calcium channel Ca_v_3.2 is removed in C-LTMRs as soon as the Nav1.8 promoter and the Cre recombinase start to be active, presumably around E16 to E17 (*25*, *26*). Thus, this “late” DRG Ca_v_3.2 cKO spare any in utero potential developmental issues, while it should start to abolish Ca_v_3.2 function at perinatal stage. If C-LTMRs were unable to detect dynamic tactile stimulation early in life, then this would have marked consequences on maternal bonding and social behavior later on and could explain the social behavior that we observed. Immunohistochemistry (IHC) analyses confirmed that, in the Ca_v_3.2^Nav1.8^cKO mouse line, Ca_v_3.2-GFP expression is mostly excluded from C-LTMRs, from birth throughout adulthood (Fig. 1, A and B, and figs. S1 to S3). However, in DRG neurons cultured from control animals 10 days after birth, we observed Ca_v_3.2-like current with a lower density to that of adult DRG neurons. With such small current, the electrogenic contribution of Ca_v_3.2 to C-LTMR excitability during early postnatal development is likely minimal. However, we cannot rule out any effects on neuronal devolvement and excitability via nonelectrogenic mechanisms related to calcium-mediated influx and protein interactions. In any case, the evidence provided here does not allow us to firmly conclude if C-LTMR function was impaired before adulthood in Ca_v_3.2^Nav1.8^cKO mice. Further in-depth investigation should be required to understand how C-LTMRs develop their excitability around birth and how Ca_v_3.2 contribute to this development.

### C-LTMRs, Ca_v_3.2, and sociability

Our results with the three-chamber social preference test indicate that Ca_v_3.2^Nav1.8^cKO mice present a social deficit, which has been confirmed using the LMT. However, thanks to the latter, we were able to better define the nature of the social interactions altered in this cKO. We observed a notable difference between passive and active social interaction between Ca_v_3.2^Nav1.8^cKO mice and their control littermates. The fact that there was no difference in passive social interactions while unilateral and reciprocal interactions were decreased suggests that Ca_v_3.2^Nav1.8^cKO mice may not avoid interindividual tactile stimuli, but may not be actively seeking for social contacts. This could be explained if C-LTMR activation contributed to reinforce social interaction through gentle dynamic stimulation, and impairing these neurons could results in the ability to reinforce social bounds through touch. Nonetheless, if these mice do not show signs of tactile avoidance, based on the parameters extracted by the LMT, it could be also due to the experimental paradigm. It may be difficult for a mouse to avoid the three other cagemates within the LMT arena.

It is also interesting to notice that Ca_v_3.2^Nav1.8^cKO mice did more rearing behavior when isolated (fig. S4D and table S1), which could be interpreted as a manifestation of anxiety (*41*). While the link between rearing and anxiety is a matter of debate, this phenotype, associated with a decreased sociability in the LMT and in the social preference test, is markedly similar to those observed in the Mecp2 and Shank3 peripherally restricted KO (*8*). In addition, Ca_v_3.2^Nav1.8^cKO mice shared some common features with the Shank2 KO mouse model of ASD, which was also phenotyped in the LMT, and which also displayed an increase of isolated behaviors and a reduction in social contacts (*30*). These observations strongly suggest that C-LTMRs are a key component of social interactions and may play a critical role in neurodevelopmental disorders such as ASD, as suggested from human observation. Individuals with ASD have altered tactile sensitivities, and autism-associated behavioral deficits and neural responses to C-LTMR–triggered affective touch stimuli are inversely correlated (*42*). Such observations suggest that people with greater numbers of autism-relevant traits may have an impaired processing of affective touch. The asocial phenotypes observed in this study in adult Ca_v_3.2^Nav1.8^cKO mice, with deficient C-LTMRs, could then be the reflection of altered bottom-up effect of touch on the social brain (*43*). Last, the asocial behavioral traits revealed here may have translational relevance in the clinic. They nicely parallel the presence of congenital missense mutations in distinct ASD patients within the *Cacna1H* gene, leading to Ca_v_3.2 functional defects (*44*). While in this case mutations have body-wide consequences, we can speculate a substantial contribution of tactile sensory deficits to explain the genotype-phenotype relations. Consequently, a perspective of developing peripherally restricted Ca_v_3.2 selective T-type calcium channel activators could represent a therapeutic opportunity to improve some deficits of ASD patients.

### C-LTMRs and pleasant touch

Using a model of chemogenetic activation of C-LTMRs, we demonstrated that this population of neurons is sufficient to create a pleasant experience. Not only does C-LTMR stimulation induced a rewarding experience in a CPP paradigm with no specific context but also it reinforced thermotaxis to 30°C, which is consistent with the idea that these neurons are tuned to the temperature of a skin-stroking caress (~30°C) similarly as in humans (*45*). Consistently, we previously documented that Ca_v_3.2^Nav1.8^cKO mice have the exact opposite phenotype with a weakened thermotaxis (*19*), and a recent study also indicated a shift in thermotaxis toward cold temperature with another model impairing the excitability of the C-LTMRs (*46*). Furthermore, the LMT experiments were performed at room temperature (RT) of 24°C, which is cool for mice. Thus, within the LMT, the reinforcement of thermotaxis by CNO evidenced in the gradient paradigm might have contributed to lead the animals to seek for warmer body temperatures in group formations. Each mouse in dyads, triads, or even tetrads may further amplify the C-LTMR activation during skin-to-skin contacts.

To our knowledge, only one study presently published has been able to demonstrate that a population of primary sensory neurons, the population expressing MRGPRB4, is able to drive a motivational behavior in mice upon DREADD stimulation approach (*47*). Unfortunately, whether this neuronal population can also drive specific interindividual social behaviors has not been investigated.

Whether the genetically defined C-LTMR population MRGPRB4 or TH/VGlut3/TAFA4 (or both) is the murine equivalent of CT fibers in humans remains controversial. The MRGPRB4-expressing population is markedly different from the TH-expressing C-LTMR population. Despite both belonging to a class of mechanoreceptive C-fibers, MRGPRB4 is expressed in a population not as well defined functionally and genetically across development than TH-expressing neurons (*27*, *48*). For example MRGPRB4-expressing neurons also express TRPV1 and respond to capsaicin (*47*) similarly to numerous C-nociceptors, whereas TH-expressing neurons do not (*23*). Humans C-tactile fibers do not seem to be sensitized by capsaicin, suggesting that they might not express TRPV1 (*7*). In addition, applying the T-type channel blocker TTA-A2 on human glabrous skin completely abolished the sensibility to innocuous tactile stimuli. This finding is supporting the expression of T-type calcium channel in C-tactile afferences, similar to mouse TH-expressing C-LTMRs, but not MRGPRB4 fibers (*19*, *49*). Together, these data strengthen our confidence that the population we defined as C-LTMRs (by using different gene expression such as TAFA4, TH, and the Ca_v_3.2-Nav1.8 tandem) is the correlate of the sensory C-tactile fibers that are supporting affective touch in humans.

### Using chemogenetics to manipulate C-LTMRs

On the basis of our IHC analysis and the changes in social interactions, the viral strategy that we developed allows to manipulate C-LTMRs with great accuracy. However, while being highly specific, it appears that the mini Ca_v_3.2 promoter used in our viral constructs may not be as efficient as other unspecific viral promoter (such as CAG, hSyn, or Ef1A). Our approach likely results in sufficient hM3Dq expression levels allowing to potentiate C-LTMR excitability rather than overexcite them, in line with the behavioral changes observed. It is important to note that we were unable to clarify if DREADD activation leads to the activation or potentiation of C-LTMRs. Since for our experiments CNO reinforced social interactions or the time spent in the comfort zone, we supposed that DREADD activation leads to C-LTMR potentiation rather than their activation. Furthermore, it has been proposed that DREADD expression in DRG can modify neuronal excitability independently of the CNO (*50*). However, even if we cannot rule out a potential effect of G protein-coupled receptor (GPCR) overexpression on neuronal excitability as described previously, the transient nature or the expression through a viral strategy is probably unlikely to induce an unbalance in cell physiology during the time of the experiment. In vitro calcium imaging in cultured DRG, from mice expressing the hM3Dq DREADD construct, confirmed that most of the neurons functionally responding to CNO had a unique C-LTMR chemo-responding pattern. This pattern was consistent with the transcriptional profile of the C-LTMR neurons regarding the expression of channels and receptors coupled to cytosolic calcium variations. This includes responses to agonists of TRPA1 as reported before (*18*, *19*), as well as to the purinergic metabotropic receptor P2RY1, which is consistently reported to be highly expressed in C-LTMRs across species from rodents to nonhuman primates (*21*, *23*, *51*, *52*). In addition, all CNO-responsive neurons where negative to live staining with fluorescent IB4, as previously reported for C-LTMRs (*18*).

### C-LTMRs as a motivational drive toward social interaction

Activation of C-LTMRs greatly increases the number of contacts between adult animals. In accordance with C-LTMR known innervation of the hairy skin, we observed a large increase of what we considered skin-to-skin contacts (contact side-by-side and side-by-side opposite, cumulative duration, and number of events; Fig. 6, D and E) (*20*). In addition, we witnessed an increase in nose-to-nose contacts and even more nose-to-anogenital contacts, involving skin areas that are not supposedly innervated by C-LTMRs, although recent publications suggest that C-LTMRs innervate more skin areas than expected in rodents, such as the glabrous skin between hind paw running pads (*46*). Such observation may be due to an increase of social investigation following a priming effect due to the activation of the C-LTMRs. A potential reinforcing influence of the C-LTMRs (by CNO and side-by-side contacts) on interindividual interaction might induce the initiation of more complex social contacts (nose-to-nose and nose-to-anogenital contacts). For example, C-LTMR stimulation during side-by-side contact may act as an appeasing signal to engage communication through nose-to-nose sniffing (*53*). A comparable effect may also explain the surprising finding that, in an LMT cage, the activation of C-LTMRs in two mice increased social interactions not only between the C-LTMR–stimulated mice but also in the control mice. Since the LMT is a closed arena, in which the four mice are free to interact with each other, changes in a given social behavior by any of the four mice will inevitably affect the social interactions of the other three mice. This may explain the alteration of interaction probability observed in control mice. In addition, it is possible that the stimulation of the C-LTMR in some mice may induce some sort of social buffering or emotional contagion, ultimately leading to positively valent social interest within the whole group, and thus increasing seeking for interindividual contact in all animals, including the controls.

In conclusion, while clinical studies documented that affective information conveyed through the skin have powerful impact on social behavior, the direct causality of C-tactile/C-LTMR primary afferents in this bottom-up regulation of the social brain remained to be demonstrated. Our study, combining mouse genetics and in-depth ethological analysis, provides a first demonstration that, in healthy naïve adults, enhancing skin C-LTMR activity for a few tens of minutes is sufficient to induce immediate prosocial effects. On the other hand, an impairment of C-LTMR functions at adulthood negatively affects sociability, with behavioral traits resembling those found in genetic mouse models of ASD. Last, the preclinical approaches that we developed and allowing to selectively control C-LTMRs will prefigure future investigations deciphering the pathophysiology of the neuronal circuits of social behaviors driven by affective touch.

## MATERIALS AND METHODS

### Study approval

All animal procedures complied with the welfare guidelines of the European Community and were approved by the local ethic comity, the Herault department Veterinary Direction, France, and the French Ministry for Higher Education, Research and Innovation (agreement number: 2017100915448101).

### Animals

Ca_v_3.2^GFP-flox^KI, Ca_v_3.2^Nav1.8^cKO (Ca_v_3.2^GFP-flox^KI x Nav1.8^cre^), and Nav1.8^cre^ mouse lines were bred and housed in a specific pathogen–free (SPF) animal facility at the Institute of Functional genomic under approved laboratory conditions (12-hour day/night cycle, 22° to 24°C, 50 ± 5% humidity, food and water ad libidum). All behavioral tests were conducted in the same animal facility with the SPF sanitary status. Experiments were performed in males because of the recent work of Bohic *et al*. (*37*), suggesting that C-LTMR impairment affects only male behavior. In addition, de Chaumont *et al*. (*30*) reported that males and females have drastic behavioral and social differences in the LMT, with female C57BL/6J mice spending more time moving and interacting, which can impaired our capacity to interpret the observation.

### Plasmids and viruses

The excitatory DREADD hM3Dq with an N-terminal HA tag epitope was cloned into a pAAV viral vector under the control of a 1.5-kb minimal Ca_v_3.2 promotor (*32*) and between double-floxed inverse orientation (also called FLEx) sequences, allowing to switch the DREADD open reading frame in the correct orientation upon Cre-mediated recombination. pAAV-promCa_v_3.2-DIO-ChR2-ires-YFP-WPRE (*32*) served as template. ChR2-ires-YFP was replaced by the HA-hM3Dq sequence, coming from pAAV-hDlx-GqDREADD-dTomato (gift from G. Fishell, Addgene plasmid #83897), using the HIFI DNA assembly method (New England Biolabs, Evry, France). A 3xHA version of the DREADD was further created by inserting a Kpn I–Nhe I cassette obtained by gene synthesis (Proteogenix, Strasbourg, France). All constructs were verified by DNA sequencing. Before viral production, the functionality of the constructs was demonstrated by Fura-2 calcium imaging following transient transfection in HEK cells together with expression vectors expressing the Cre recombinase (pCAG-Cre-ires-GFP, a gift from J. Dujardin) and the Egr1 transcription factor (pcDNA3-Egr1, a gift from E. Adamson, Addgene plasmid #11729) known to stimulate the Ca_v_3.2 promotor (*34*).

AAV_PHPs_ viruses were custom-made either by the Plateforme de Vectorologie de Montpelier (Montpellier, France) or by the Canadian Neurophotonics Platform Viral Vector Core Facility (RRID:SCR_016477) (Quebec, Canada) using the ad hoc capsid plasmid [pUCmini-iCAP-PHP.S, a gift from V. Gradinaru, Addgene plasmid #103006; (*33*)]. Control rAAV PHP CAG mCherry was bought from Addgene (viral prep #59462-PHP.S).

### In vivo DRG single-unit recordings

Mice were anaesthetized with urethane (20%, 1.5 mg/kg) and placed on a stereotaxic frame with the vertebral column stabilized by vertebral clamps to ensure reliable electrophysiological recordings. Body temperature was maintained by a heated blanket throughout the experiment. A laminectomy was performed on lumbar vertebrae L3 to L5, and L4 DRG was exposed after removal of the vertebral bone. The ipsilateral sciatic nerve was isolated, mounted on stimulating electrodes, covered with paraffin oil, and connected to a DS3 stimulator (Digitimer Ltd., UK). Extracellular recordings of DRG neurons were performed using borosilicate glass capillaries (2mΩ, Harvard Apparatus, USA) filled with NaCl (4%). Data were acquired using a CED 1401 interface (Cambridge Electronic Design, UK) and stored on a computer. The responses to both brush and pressure stimuli were characterized in the most responsive part of the skin receptive field with a marten hair brush and von Frey filaments (ranging from 0.008 to 10 g), respectively. Data were analyzed offline with Spike2 software (Cambridge Electronic Design, UK). In each experiment, specifically responding single unit was characterized by its size and shape. On the basis of the latency measured between the spike and the artifact following electrical stimulation of the sciatic nerve, a CV was assigned to each single unit. These were considered either as fast or slow conducting single unit whether the latency was lower and higher than 30 ms, respectively.

### Primary sensory neuron culture, electrophysiology, and calcium imaging

Lumbar/thoracic DRGs (L4 to T10) were prepared and cultured on laminin-coated μ-Dish chambers (Ibidi, Germany) as described previously (*19*) from Nav1.8^Cre^ mice injected with AAV_PHPs_-pCa_v_3.2-FLEx-HA-hM3Dq 18 weeks before the experiment. Recordings were made after 12 hours of culture. To identify GFP^+^ and IB4^−^ neuron, anti-GFP rabbit (1:200; Chromotek) antibody was incubated for 10 min in culture medium, followed by incubation of anti-rabbit Alexa Fluor 488 antibodies (1:500) and IB4 conjugated to Alexa Fluor 596 (1:2000) for 10 min in culture medium as well. After three quick washes in recording solution, the cells were recorded.

For calcium current recordings, the extracellular solution contained 2 mM CaCl_2_, 100 mM TEACl, 2 mM NaCl, 1 mM MgCl_2_, 40 mM choline Cl, 5 mM glucose, 5 mM 4AP (pH 7.4 with TEAOH, ~330 mOsM). Pipettes with a resistance of 1 to 1.5 megohms were filled with an internal solution containing 110 mM CsCl, 3 mM MgCl_2_, 10 mM EGTA, 10 mM Hepes, 3 mM Mg–adenosine triphosphate, and 0.6 mM guanosine triphosphate (pH 7.4 with CsOH, ~300 mOsM). Currents were recorded using an Axopatch 200B amplifier. Recordings were sampled at 8 kHz and filtered at 10 kHz. Data were analyzed by pCLAMP10 (Molecular Devices). Current-voltage curves (*I*-*V* curves) were fitted using a combined Boltzmann and linear ohmic relationships.

For calcium imaging, before recording, neurons were incubated with 5 μM Fura-2 acetoxymethyl (AM) in Tyrode’s solution for 1 hour at 37°C and then washed and kept for an additional 20 min in Tyrode with no Fura-2 for deesterification. Fluorescence measurements were sampled at 1 Hz with an inverted microscope (Olympus IX70) equipped with an Evolves Photometrics electron-multiplying charge-coupled device camera (Roper Scientific, France). Fura-2 was excited at 340 and 380 nm, and ratios of emitted fluorescence at 510 nm were acquired using MetaFluor software (Universal Imaging). Drugs were applied with a gravity-driven perfusion (1 to 2 ml/min). Pharmacological agonists of hM3Dq (CNO, 30 μM; Tocris, France), P2YR1 (MRS, 200 nM; Tocris France), TRPV1 (capsaicin, 500 nM), TRPA1 (AITC, 200 μM), and KCl (40 mM) were prepared into the Tyrode solution and applied sequentially to the neurons for a few seconds. Data were analyzed offline using MetaFluor, Excel, and GraphPad. Similar procedure for Fura-2 loading and imaging was used for transiently transfected HEK cells for the verification of pAAV functionality before custom virus production. Otherwise stated, all chemicals were from Sigma-Aldrich (L’isle d’Abeau Chesnes, France).

### Intrathecal injection

Six- to 8-week-old mice were anesthetized by inhalation of a 2% isoflurane/1.5% oxygen mixture. Five microliters of viral vector (1 × 10^13^) was injected into the lumbar subarachnoid space using a 5-μl Hamilton syringe and a glass capillary adaptor. Wounds were sutured, and surgical sites were infiltrated with 2% lidocaine in saline. Animals were placed on a heating pad in an oxygenated chamber and monitored until fully recovered.

### Behavior

#### Three-chamber social preference test

Eight- to 12-week-old male mice were first habituated for half an hour to the experimental room in their home cage. The three-chamber arena is composed of three compartment boxes, each 20 × 40 × 22 (h) cm, and separated by two sliding doors [5 × 8 (h) cm], with one prison placed in the upper right corner of the right compartment and another one on the lower left corner of the left compartment. After room habituation, each animal was placed individually in the arena, starting from the middle compartment, and was let free to explore for 10 min with the two prisons empty. Then, the mouse was locked in the middle compartment and one mouse, stranger to the tested mouse (C57B6J/n bred and housed in the IGF facility), was placed in one of the two prisons, and an inanimate object (made from Lego blocks, roughly shaped like a mouse) was placed in the other prison. The tested mouse was then set free to explore all compartments again for 10 min. The whole experiment was video-recorded, and animal position was tracked using EthoVision XT13 (Noldus, The Netherlands). The preference index was calculated with the following formula (Time in stranger side − Time in object side)/exploration time. A mouse was considered interacting automatically by EthoVision when the tested animal was facing the middle of the prison inside a 1-cm perimeter around the prisons. The side where the stranger mouse and the inanimate object were placed was alternated between each tested mouse to avoid bias. Experiments were performed in the morning (from 7 a.m. to 10 a.m.) under 180 lumens. The experimenter was blinded to the animal conditions.

#### Live Mouse Tracker

The LMT setup was built following the instruction of de Chaumont *et al*. (*30*). We added to the original design two water dispensers to the arena on two sides.

Before going into the LMT, each group of four mice was housed together for at least 2 weeks [following Radio Frequency Identification (RFID) chip implantation under anesthesia]. LMT recordings were performed with 10- to 16-week-old Ca_v_3.2^GFP-flox^KI or Ca_v_3.2^GFP-flox^KI × Nav1.8^cre^ mice, and with 14- to 18-week-old Nav1.8^cre^ mice (injected with rAAV_PHPs_-CAG-mCherry or rAAV_PHPs_-Ca_v_3.2-FLEx-HA-hM3Dq). During recording, the animals were kept under the same condition as in the housing facility (12-hour daylight, 500 lumens, food and water *ad libidum*) and the experimenter came once per day to perform injection if necessary or to check the water and food level.

##### Active (reciprocal and unilateral) and passive interaction definition

Similarly to published work (*54*), we defined active (reciprocal and unilateral) and passive social interactions based on the localization of the social contact on the body of the two mice interacting. We used the automatically annotated event extracted by the LMT and considered all contacts associated with nose-to-nose contacts and side-by-side contacts as reciprocal interaction. Unilateral social interactions were associated with giving ano-genital contacts (subject is contacting the rear of another mouse with its nose), and passive social interaction was associated with receiving ano-genital contacts (subject rear is being touched by the nose of another mouse).

We used Python scripts provided to analyze all the data acquired by the system. (https://livemousetracker.org/; https://github.com/fdechaumont/lmt-analysis). We only performed analysis during the activity phase (night cycle) as during the day mice nest together, which impaired the tracking and provided unreliable annotation.

#### Von Frey

Eight weeks after intrathecal injections, mice were first habituated for half an hour to the experimental room in their home cage. Each mouse was then placed individually into small arena (8 × 8 cm) over a von Frey mesh for 45 min for habituation. Then, animals were injected peritoneally with CNO (1 mg/kg) diluted in sterile saline (prepared fresh each time). Thirty minutes to 45 min after the injections, von Frey filaments (0.07, 0.6, and 2 g) and the brush were applied five times on each hind paw and withdrawal was then scored. Experiments were performed in the morning (from 8 a.m. to 11 a.m.) under 500 lumens. The experimenter was blinded to the animal conditions.

#### Temperature gradient

Eight weeks after intrathecal injections, mice were first habituated for half an hour to the experimental room in their home cage and then injected with CNO (1 mg/kg) diluted in sterile saline (prepared fresh each time). Thirty minutes later, mice were placed into the Bioseb thermal gradient (two 1m50 long corridors, with one extremity cooled down to 5°C and the other heated to 50°C, creating a thermal gradient spited into 20 thermal zones). Mice were free to explore for 90 min during which animal’s positions were tracked and annotated accordingly to the temperature zones. For each run, a C-LTMRs^hM3Dq^ mouse and a control mouse were tested simultaneously. However, the experimental groups were labeled as such as the experimenter was still blind to the animal conditions. Mice corridor distribution was alternated between each run to avoid any corridor biased. Experiments were performed in the morning (from 8 a.m. to 12 a.m.) under 180 lumens.

#### Conditioned place preference

Eight weeks after intrathecal injections, mice were subjected to the CPP protocol. This protocol consisted of six experimental days: The first day consisted of an exploration and habituation phase of 30 min per mouse, where mice were free to explore the CPP arena (compartments: 25 cm by 20 cm, corridor: 5 cm by 20 cm). From the second to the fifth day, animals were conditioned to associate a saline injection in the morning to their preferred compartment (defined during the first day) and a CNO injection (1 mg/kg) to the other compartment in the afternoon. During this conditioning, animals received the injection and stayed in their homecage for 30 min and then were placed into the designated compartment for 30 min to allow the CNO to reach its molecular target. To avoid that the CNO was still active between two conditionings, we choose to stretch as much as possible the period of time between the CNO injection from one given day and the next day saline injection. On the sixth and last day, animals were free to explore the whole arena again for 30 min. The position of the animal was detected by a network of infrared beams. The CPP rack allowed us to perform four experiments at a time (Imetronic, Marcheprime, France); thus, each run was balanced to test two C-LTMRs^hM3Dq^ and two control mice. However, the experimental groups were labeled as such as the experimenter was blinded to the animal conditions. Experiments were performed in the morning (from 8 a.m. to 12 a.m.) under 180 lumens.

### Immunohistology

#### Tissue collection and processing

Adult mice were transcardially perfused with phosphate-buffered saline (PBS) followed by 10% formaldehyde in PBS. Brain and spinal cord were dissected, postfixed in 10% formaldehyde for 24 hours, and cryoprotected in 30% sucrose in PBS. DRGs were cryoprotected in 30% sucrose in PBS directly after formaldehyde perfusion. For P0 and P10, animals were anesthetized on ice before euthanasia by beheading. For P0, the entire spine was collected and postfixed in 10% formaldehyde for 24 hours, and DRGs were then dissected out and cryoprotected in 30% sucrose in PBS. For P10, DRGs were collected in cold PBS, postfixed for 1 hour at RT in 4% paraformaldehyde, and then cryoprotected in 30% sucrose in PBS. Tissues were then frozen in optimum cutting temperature (Tissue-Tek) and sectioned using a cryostat (Leica). Spinal cord and brain were sectioned at 40 μm and stored in PBS + 0.05% azide at 4°C. For DRGs, tissues were sectioned at 18 μm, collected on Superfrost Plus slides (Fisher Scientific), and stored at −80°C.

#### Immunofluorescence

Tissues were incubated for 1 hour and blocked in a solution consisting of 0.1 M PBS with 0.3% Triton X-100 (Sigma-Aldrich) plus 5% normal donkey serum. Primary and secondary antibodies were diluted in 0.1 M PBS with 0.3% Triton X-100 plus 1% normal donkey serum. Sections were then incubated overnight at 4°C in primary antibody solution, washed in 0.1 M PBS with 0.3% Triton X-100 for 40 min, incubated for 2 hours in secondary antibody at RT, and washed again in 0.1 M PBS for 40 min. Sections were then mounted using Dako fluorescence mounting medium. Images were acquired with a Leica SP8 confocal microscope. For experiments regarding AAV_PHPs_-pCa_v_3.2-FLEx-HA-hM3Dq validation, for each mouse, three sections from S1, L3, and T8 DRGs were analyzed and counted.

Primary antibodies: The following antibodies were used: anti-TH: Millipore (sheep; 1:500), anti–HA tag: Covenant (mouse, 1:1000), anti-Vglut3: Synaptic Systems (rabbit, 1:500), anti-TrkA: Millipore (rabbit, 1:500), anti-TrkB: R&D Systems (goat, 1:500), anti-NF200: Aves (chicken, 1:200), anti-ret: R&D Systems (goat, 1:250), anti-GINIP: gift from A. Moqrich (rat, 1:2000), and anti-CGRP: Peninsula (rabbit, 1:200). To identify IB4-binding cells, fluorophore-conjugated IB4 (1:500; Vector Laboratories) was used in place of primary and secondary antibodies. Secondary antibodies: Alexa Fluor–conjugated secondary antibodies were acquired from Invitrogen and Jackson ImmunoResearch Labs.

### Statistics

Statistics were performed with GraphPad Prism 8. Normality was tested using the D’Agostino-Pearson test. Statistical tests used to compare the different data points are indicated in the figure legends. Data are represented as means ± SEM. For bar graphs, individual data points were superimposed under means ± SEM.

## Notes

View/request a protocol for this paper from *Bio-protocol*.
