# Supplementary material for: The impact of C-tactile low-threshold mechanoreceptors on affective touch and social interactions in mice
Source: Sci Adv. 2022 Jun 29;8(26):eabo7566. doi: 10.1126/sciadv.abo7566 (PMC9242590; doi:10.1126/sciadv.abo7566)
Supplement: Supplementary file 1 — Figs. S1 to S9 [file sciadv.abo7566_sm.pdf]

Science Advances
AAAS

Supplementary Materials for

The impact of C-tactile low-threshold mechanoreceptors on affective touch and social interactions in mice

Damien Huzard *et al.*

Corresponding author: Amaury François, amaury.francois@igf.cnrs.fr

Sci. Adv. **8**, eabo7566 (2022)
DOI: 10.1126/sciadv.abo7566

The PDF file includes:

Figs. S1 to S9
Legends for tables S1 and S2

Other Supplementary Material for this manuscript includes the following:

Tables S1 and S2

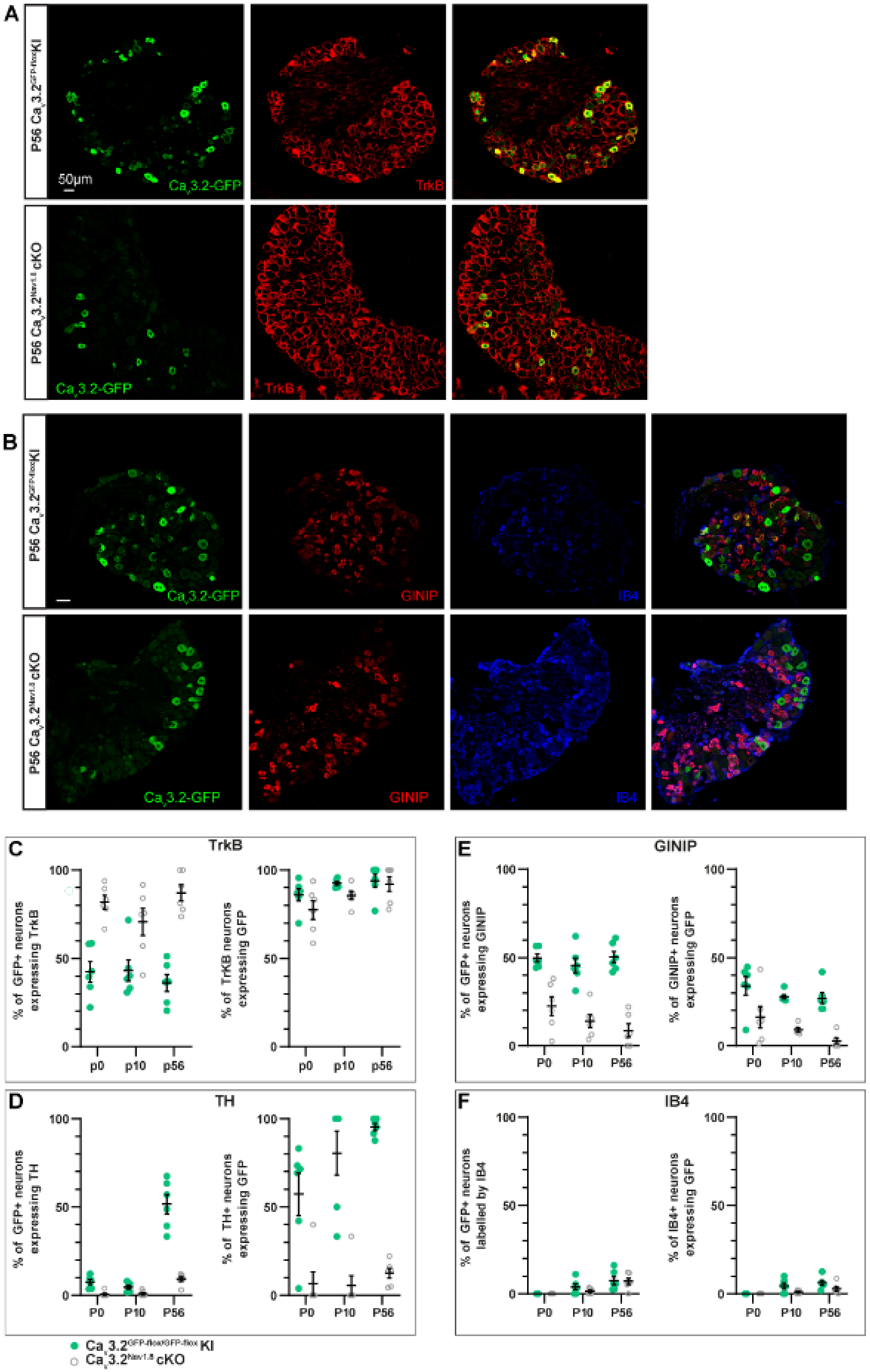
A
P56 Cav3.2GFP-flox KI
50µm
Cav3.2-GFP
TrkB
P56 Cav3.2Nav1.8 cKO
Cav3.2-GFP
TrkB
B
P56 Cav3.2GFP-flox KI
Cav3.2-GFP
GINIP
IB4
P56 Cav3.2Nav1.8 cKO
Cav3.2-GFP
GINIP
IB4
C
TrkB
% of GFP+ neurons expressing TrkB
% of TrkB neurons expressing GFP
p0
p10
p56
D
TH
% of GFP+ neurons expressing TH
% of TH+ neurons expressing GFP
P0
P10
P56
E
GINIP
% of GFP+ neurons expressing GINIP
% of GINIP+ neurons expressing GFP
F
IB4
% of GFP+ neurons labelled by IB4
% of IB4+ neurons expressing GFP
Cav3.2GFP-flox/GFP-flox KI
Cav3.2Nav1.8 cKO

Supplementary Figure 1. $Ca_v3.2$ expression at P56 in $Ca_v3.2^{GFP\text{-}flox}$KI and $Ca_v3.2^{Nav1.8}$cKO

Representative images of P56 DRG of $Ca_v3.2$ (GFP) in green combined with TrkB in red (**A**), or GINIP in red and IB4 in blue (**B**). For each panel, the top images are representative of $Ca_v3.2^{GFP\text{-}flox}$KI and the bottom images are representative of $Ca_v3.2^{Nav1.8}$cKO. The white bar indicates the scale: 50µm.

(**C**) to (**F**) Quantification of the percentage of GFP co labelling with the different marker TrkB (**C**), TH (**D**), GINIP (**E**) or IB4 (**F**) at P0, P10, P56. Left graph: percentage of GFP neurons that also express the given marker. Right graph: percentage of neurons expressing the given marker also expressing the GFP. Light green circles: $Ca_v3.2^{GFP\text{-}flox}$KI open black circles: $Ca_v3.2^{Nav1.8}$cKO. Each dot represents one mouse (3 sections counted per mice). n = 6 per condition.

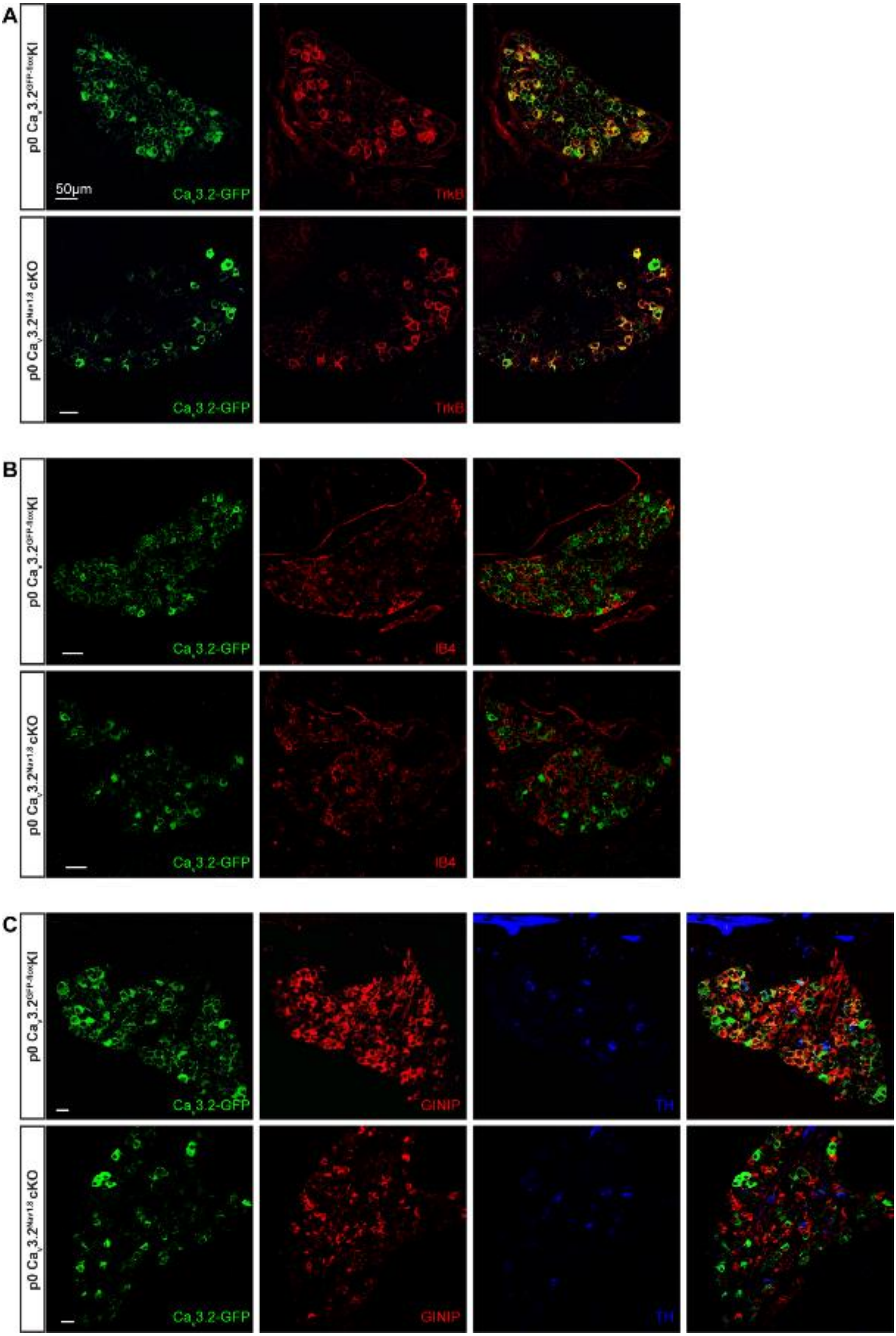
A
p0 Cav3.2GFP-floxKI
50µm
Cav3.2-GFP
TrkB
p0 Cav3.2Nav1.8 cKO
Cav3.2-GFP
TrkB
B
p0 Cav3.2GFP-floxKI
Cav3.2-GFP
IB4
p0 Cav3.2Nav1.8 cKO
Cav3.2-GFP
IB4
C
p0 Cav3.2GFP-floxKI
Cav3.2-GFP
GINIP
TH
p0 Cav3.2Nav1.8 cKO
Cav3.2-GFP
GINIP
TH

Supplementary Figure 2. $Ca_v3.2$ expression at P0 in $Ca_v3.2^{GFP\text{-}flox}$KI and $Ca_v3.2^{Nav1.8}$cKO mice. Representative images of P0 DRG of $Ca_v3.2$ (GFP) in green combined with TrkB in red (**A**), or IB4 in red (**B**), or GINIP in red and TH in blue (**C**). For each panel, the top images are representative of $Ca_v3.2^{GFP\text{-}flox}$KI and the bottom images are representative of $Ca_v3.2^{Nav1.8}$cKO. The white bar indicates the scale: 50µm.

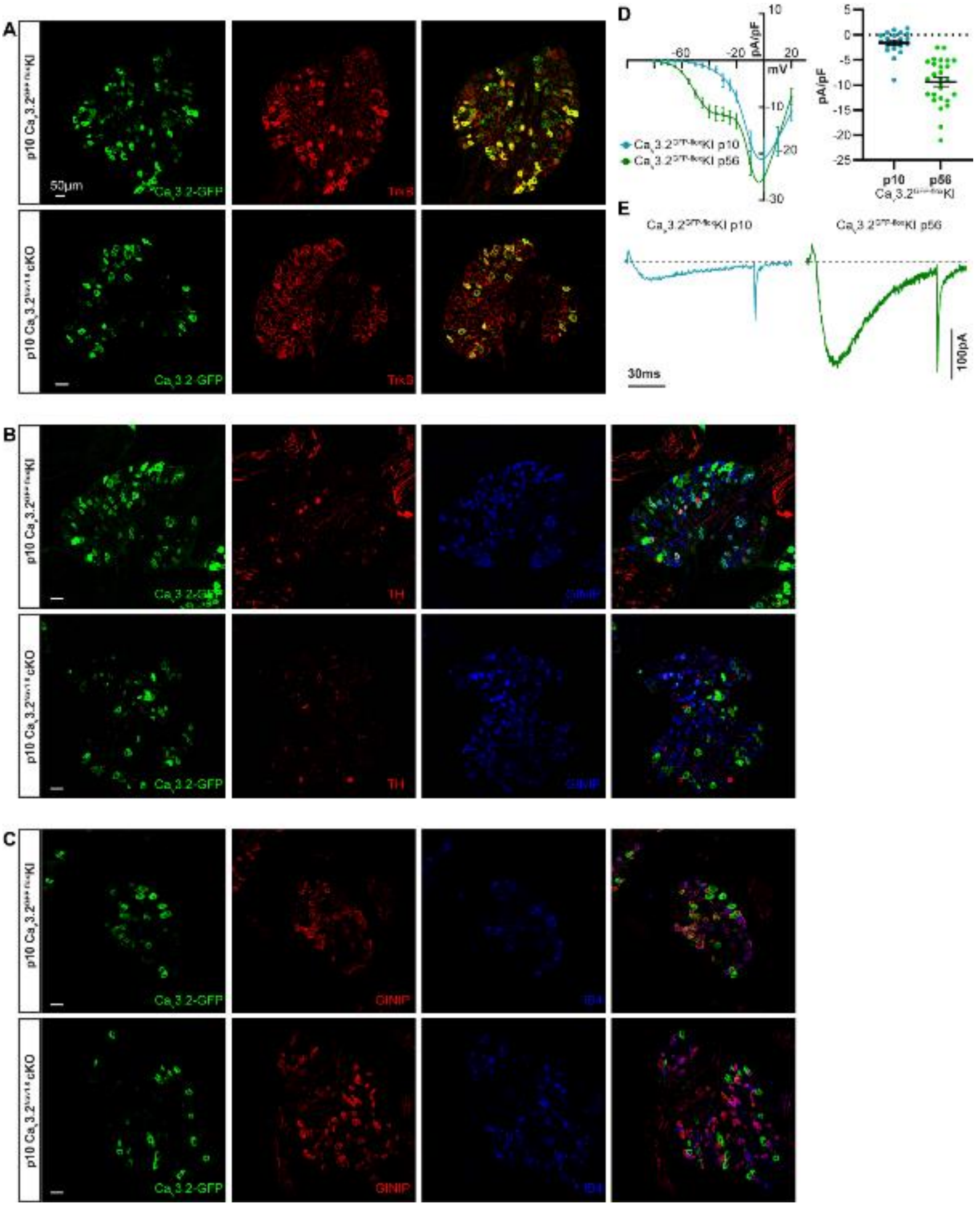

Supplementary Figure 3. $Ca_v3.2$ expression at P10 in $Ca_v3.2^{GFP\text{-}flox}$ and $Ca_v3.2^{Nav1.8}$cKO mice. Representative images of P10 DRG of $Ca_v3.2$ (GFP) in green combined with TrkB in red (**A**), or TH in red and GINIP in blue (**B**) or GINIP in red and IB4 in blue (**C**). For each panel, the top

images are representative of $Ca_v3.2^{GFP\text{-}flox}$KI mice and the bottom images are representative of $Ca_v3.2^{Nav1.8}$cKO animals. The white bar indicates the scale: 50µm.

(**D**) Left panel: IV curve at holding potential (HP) of -90mV for low and high voltage-activated calcium currents in small diameter GFP+/ IB4- DRG neurons from $Ca_v3.2^{GFP\text{-}flox}$KI mice at P10 (Blue, n=22 from 6 animals) and P56 (Green, n=29 from 5 animals). Right: Low voltage activated calcium current density at -40mV (HP-90mV) for $Ca_v3.2^{GFP\text{-}flox}$KI positive DRGs at P10 (Blue) and P56 (Green).

(**E**) Low voltage-activated calcium current examples at -40mV (HP -90mV) from P10 (Blue) and P56 (Green) $Ca_v3.2^{GFP\text{-}flox}$KI.

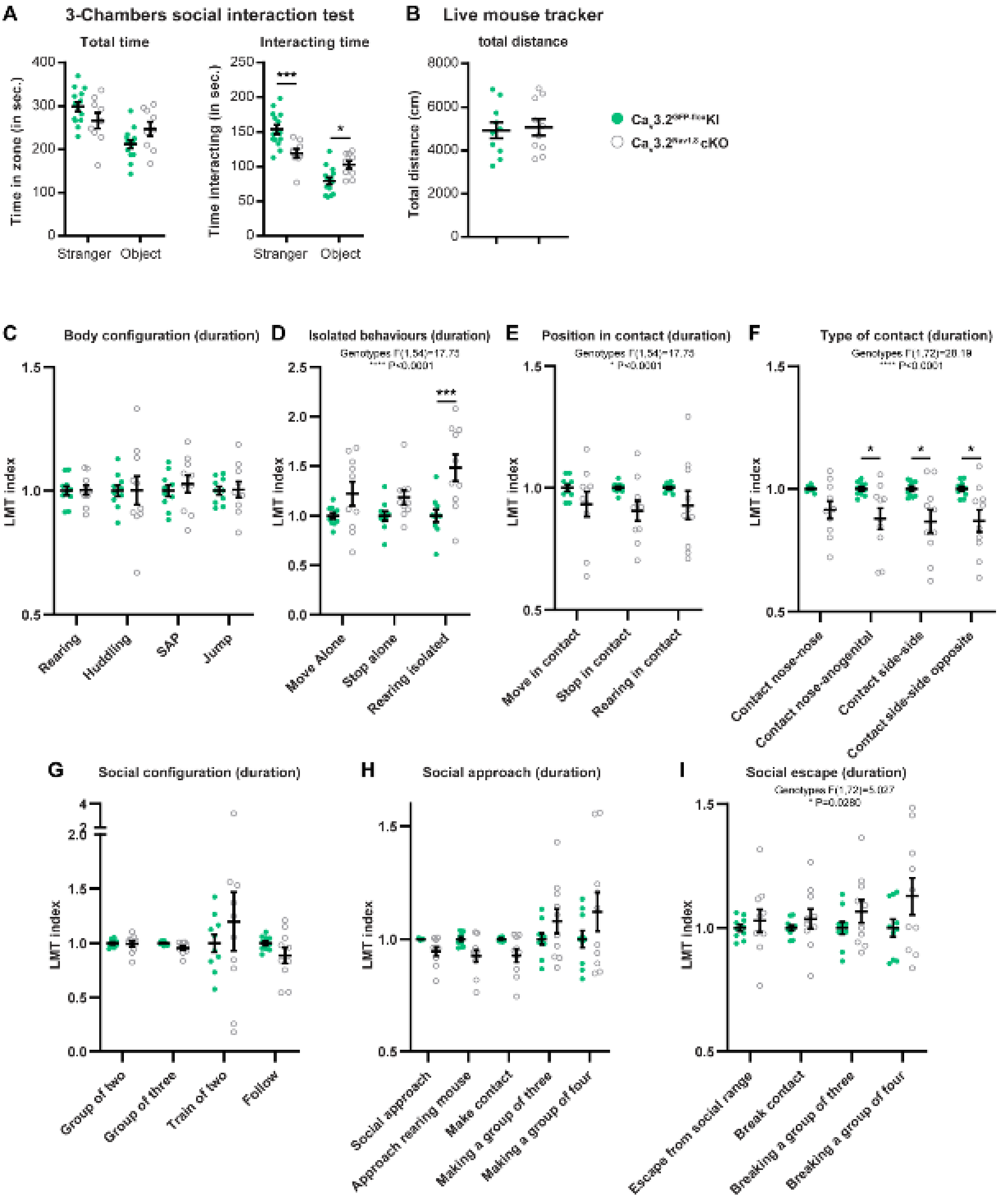

Supplementary Figure 4. C-LTMRs hypo excitability via Ca$_v$3.2 deletion reduces social behavior.

(**A**) In a three-chamber social interaction test, control Ca$_v$3.2$^{GFP\text{-}flox}$KI littermates spent more time interacting with a stranger mouse and less time with an unanimated object than Ca$_v$3.2$^{Nav1.8}$cKO. 2-way ANOVA with Bonferroni post-hoc test, ***p = 0.0007; *p = 0.0262; Ca$_v$3.2 $^{GFP\text{-}flox}$KI, n = 14 (light green circles) and Ca$_v$3.2$^{Nav1.8}$cKO, n = 9 (open grey circles).

(**B**) Sum of the total distance travelled by each mouse during the three nights for $Ca_v3.2^{GFP\text{-}flox}KI$ (light green circles, n = 10) and $Ca_v3.2^{Nav1.8}cKO$ (open grey circles, n = 10).

(**C**) to (**I**) LMT indexes of $Ca_v3.2^{GFP\text{-}flox}KI$ (light green circles; n = 10) and $Ca_v3.2^{Nav1.8}cKO$ (open grey circles; n=10) for the duration of each behaviors annotated by the LMT grouped in behavioral categories: Body configuration (**C**), Isolated behaviors (**D**), position in contact (**E**), Type of contact (**F**), Social configuration (**G**), Social approach (**H**) and social escape (**I**). Each dot represents one mouse. 2-way ANOVA with Sidak post-hoc test. ANOVA results are indicated below each panel title and full results and statistics are presented in **Table 1**.

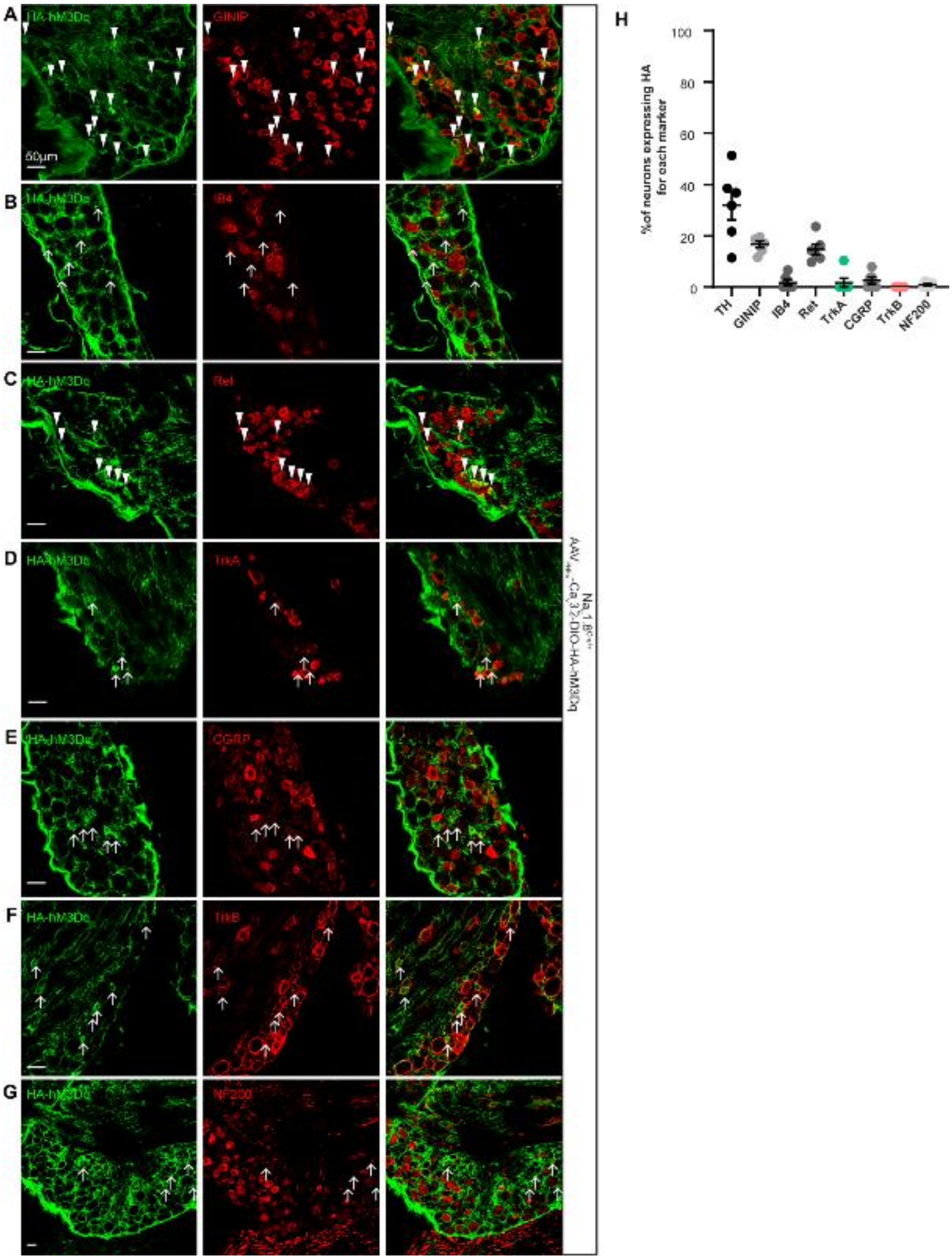

Supplementary Figure 5. Immunohistochemistry validation of the viral construct used to target C-LTMRs.

(**A**) to (**G**) representative images of a double Immunofluorescence labelling of the HA tag in green (HA-hM3Dq) with in red, either GINIP (**A**), IB4 (**B**), Ret (**C**), TrkA (**D**), CGRP (**E**), TrkB

(**F**) and NF200 (**G**). Filled arrowheads indicate examples of neurons positive for HA and one of the different markers. Arrows indicate examples of neurons positive for HA only.

(**H**) Summary of the percentages of neurons expressing HA for each marker displayed in panels (**A-G**). Each dot represents one mouse (3 sections counted per mice). n = 6.

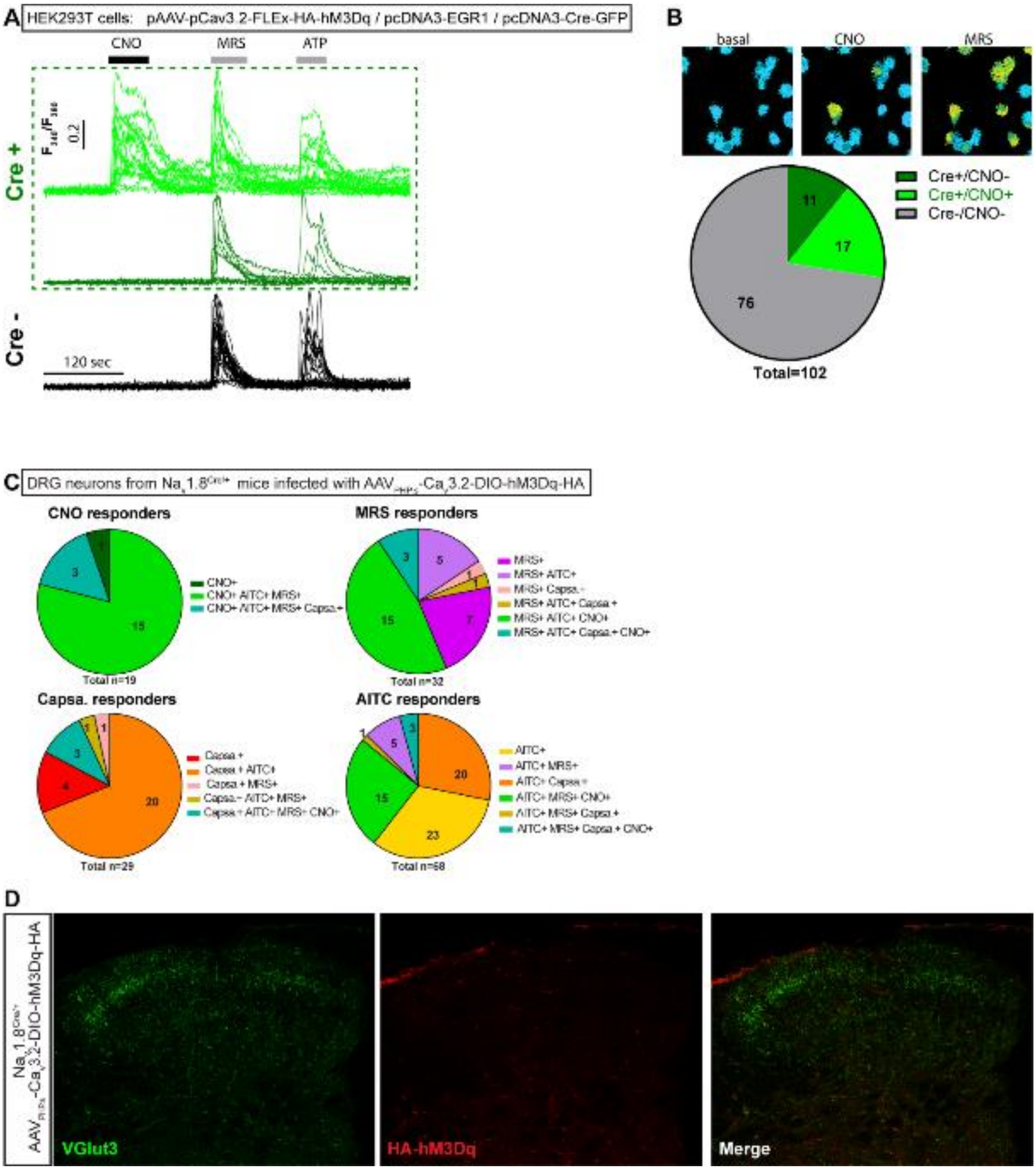

Supplementary Figure 6. *In vitro* validation of the viral construct used to target C-LTMRs.

(**A**) Representative individual Fura-2 calcium influx in cultured HEK293T cells transfected with pAAV-pCa$_V$3.2-FLEx-HA-hM3Dq, pcDNA3-EGR1, and with (Cre+, green) or without (Cre-, black) pcDNA3-Cre-GFP, following bath perfusion of CNO (30µM), MRS2365 (MRS, 200nM) to stimulate endogenous P2Y1 receptors, or ATP (30µM) to stimulate all endogenous P2Y receptors. Green traces: cells expressing the Cre recombinase (visualized with the GFP). Black traces: cells that do not express the Cre recombinase.

(**B**) Top panels, representative images of ratiometric calcium imaging on HEK293T cells transfected with pAAV-Ca_v3.2-FLEx-HA-hM3Dq, pcDNA3-EGR1, and with (Cre+) or without (Cre-) pcDNA3-Cre-GFP following bath perfusion of CNO to stimulate hM3Dq (30µM, middle image), and MRS2365 (200nM, right image). Bottom panel: Pie chart of the number or cells responding to CNO perfusion and expressing the Cre-GFP (Cre+) or not (Cre-). In total, 102 cells were recorded.

(**C**) Pie charts of the number of DRG neurons from $Na_v1.8^{Cre/+}$ mice infected with AAV_{PHPs}-pCa_v3.2-FLEx-HA-hM3Dq responding to the different chemical compounds. Based on the amplitude of the calcium influx responses, neurons were classified as CNO responder (Top Left), MRS responder (Top right), Capsaicin responder (bottom left) and AITC responders). A total of 168 neurons were recorded from 3 animals.

(**D**) Representative images of VGlut3 (Green) and HA (Red) immunostaining in the caudal part of the lumbar spinal cord.

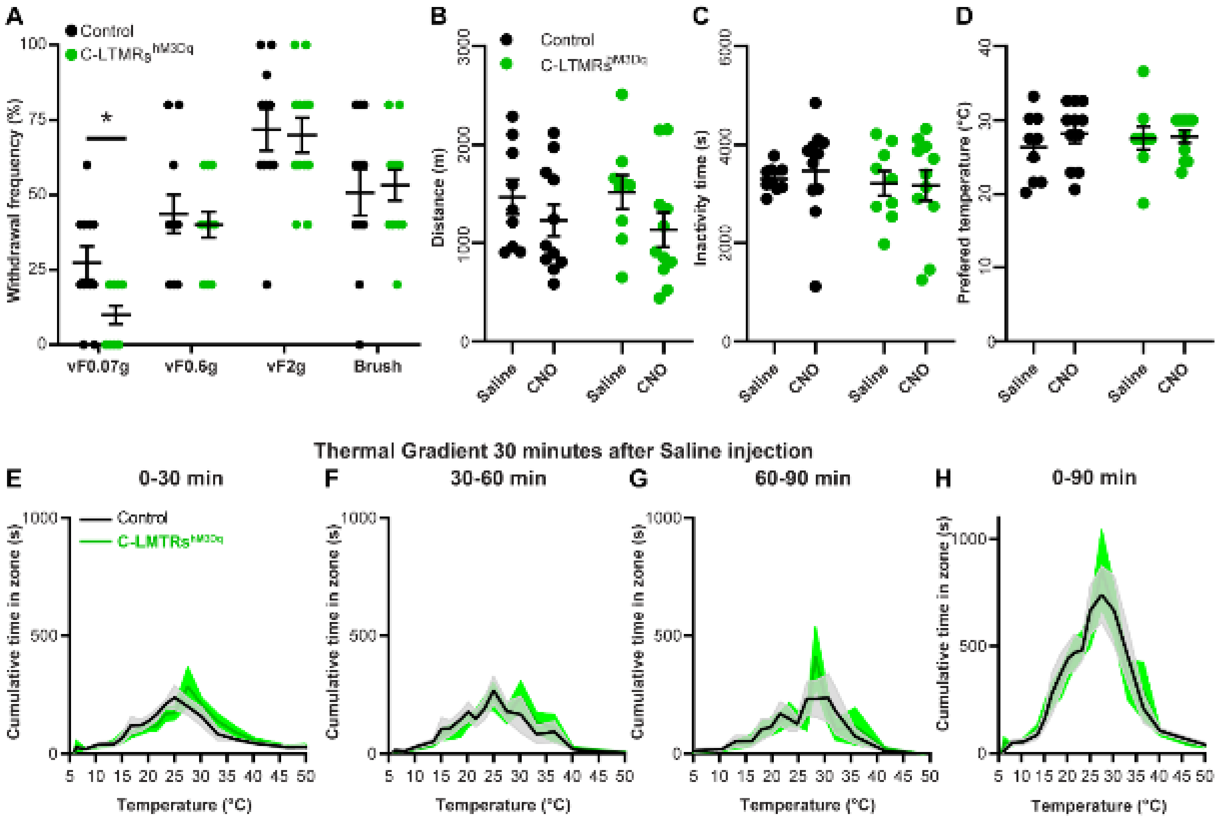

Supplementary Figure 7. Light touch perception is altered by C-LTMRs exogenous activation.

(**A**) Withdrawal frequency to von Frey filament 0.07g, 0.6g and 2g of Control ($Na_v1.8^{Cre}$; AAV_{PHPs}-CAG-mCherry; in black) and C-LTMRs^{hM3Dq} ($Na_v1.8^{Cre}$; AAV_{PHPs}-pCa$_v$3.2-FLEx-HA-hM3Dq; in green) mice. Mann-Whitney test; *p = 0.0221; Control, n = 11 and C-LTMRs^{hM3Dq}, n = 12)

(**B**) Distance travelled during 90 minutes on the thermal gradient after Saline or CNO injection.

(**C**) Cumulative time of inactivity during 90 minutes on the thermal gradient after Saline or CNO injection.

(**D**) Average temperature where the animals spent the most of their time after Saline or CNO injection.

(**E**) to (**F**) results obtained on the thermal gradient 30 min after IP injection of saline

(**E**) Cumulative time in the thermal gradient zones during the first 30 minutes after Saline injection.

(**F**) Cumulative time in the thermal gradient zones between 30 and 60 minutes after Saline injection.

(**G**) Cumulative time in the thermal gradient zones between 60 and 90 minutes after Saline injection.

(**H**) Cumulative time in the thermal gradient zones for the entire 90 minutes after Saline injection.

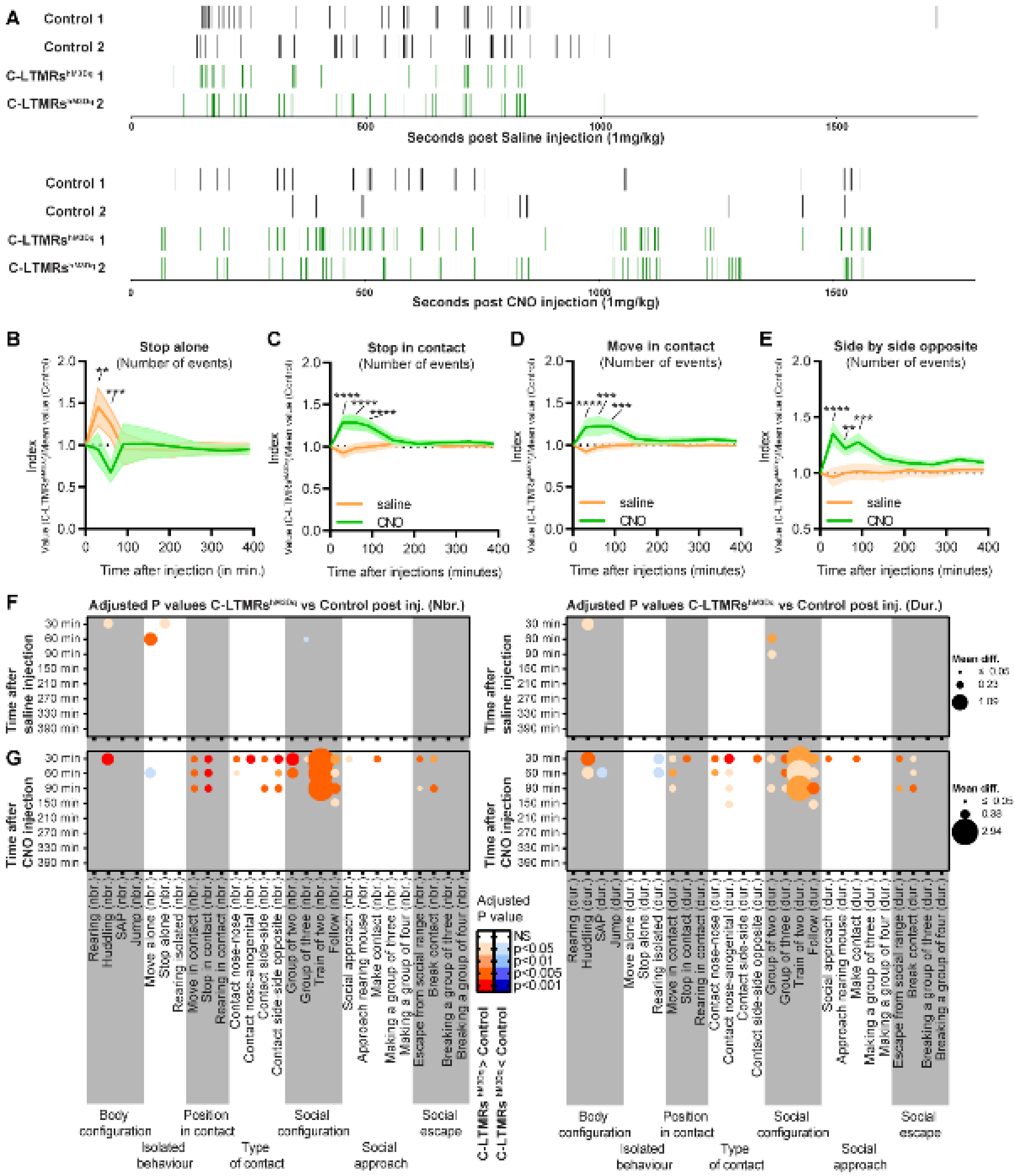

Supplementary Figure 8. C-LTMRs exogenous activation transiently increases social exploratory contacts between animals.

(**A**) Example from the 4th group of nose-to-nose contacts events for the first 30 minutes post-saline (upper panel) or CNO (lower panel) injections.

(**B**) to (**E**). LMT index obtained at 30, 60, 90, 150, 210, 270, 330, and 390 minutes post-saline injection (orange) or post-CNO injection (green) for the cumulative number of events stop alone (**B**), stop in contact (**C**), move in contact (**D**), or side by side opposite contacts (**E**). 2-way RM

ANOVA, LMT index C-LMTRs^{hM3Dq} CNO vs Saline. Sidak post-hoc test, *$p < 0.05$; **$p < 0.01$; ***$p < 0.005$; ****$p < 0.0001$.

(**F**) and (**G**) Representation of the adjusted P value and the mean difference obtained by comparing the LMT indexes from Control vs C-LMTRs^{hM3Dq} after Saline (**F**) or CNO (**G**) injections at all the different time point. 2-way RM ANOVAs performed on each time course for every number (Left) and duration (right) of events for each behavioral trait automatically annotated by the LMT. Sidak post-hoc test. The mean differences were indicated by the size of the dots and the P values were color-coded depending on the level of significance and the polarity of the mean difference used for comparison: Mean C-LMTRs^{hM3Dq} > mean Control (shade of red) or Mean C-LMTRs^{hM3Dq} < Mean Control (shade of blue) $n = 10$. All the p-values, confidence intervals, and mean differences are indicated in **Table 2.**

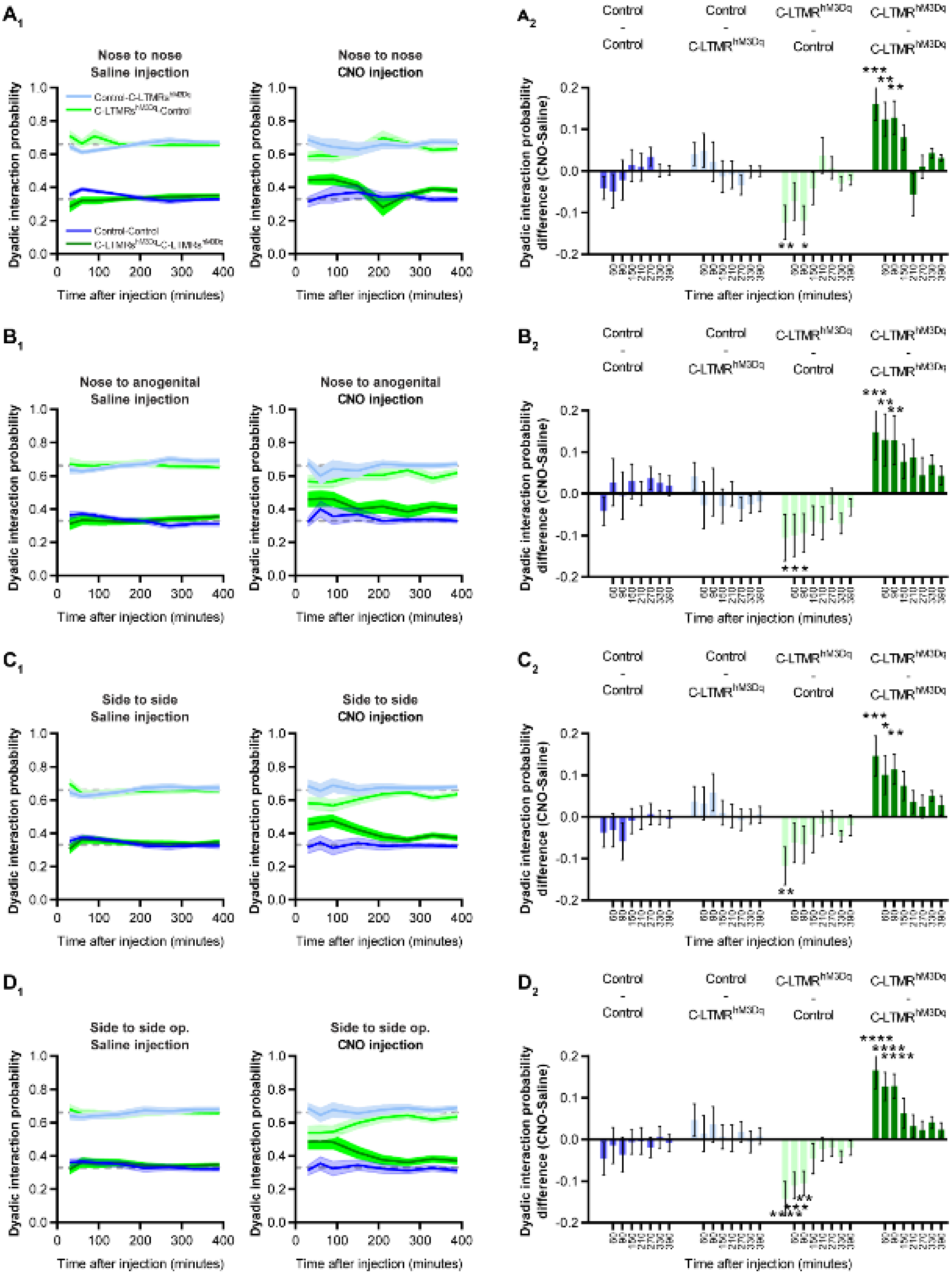

Figure Supplementary 9: C-LTMRs exogenous activation exacerbates interaction probabilities between C-LTMR^{hM3Dq} mice.

(**A$_1$**) to (**D$_1$**). Specific dyadic interaction probability for all 4 possibilities at 30, 60, 90, 150, 210, 270, 330, and 390 minutes after saline (left) and CNO (right) injection. (**A$_1$**) nose to nose dyadic

interaction; (**B_1**) nose to anogenital dyadic interaction; (**C_1**) side to side dyadic interaction; (**D_1**) side to side opposite dyadic interaction.

(**A_2**) to (**D_2**). Specific dyadic interaction probability difference between CNO and saline injections at 30, 60, 90, 150, 210, 270, 330, and 390 minutes post-injection. (**A_2**) nose to nose dyadic interaction; (**B_2**) nose to anogenital dyadic interaction; (**C_2**) side to side dyadic interaction; (**D_2**) side to side opposite dyadic interaction.

Asterisks referred to the comparison between difference at baseline (T-1hour before injection) and the difference for each time points for each group. two-way RM ANOVA, Sidak post-hoc test, *$p < 0.05$; **$p < 0.01$; ****$p < 0.0001$.

Table S1: Raw values and details from the statistical analyzes of the LMT results for $Ca_v3.2^{GFP\text{-}flox}$KI and $Ca_v3.2^{Nav1.8}$cKO mice.

Table S2: Raw values and details from the statistical analyzes of the LMT results for C-LTMR^{hM3Dq} and Control animals, after saline or CNO injections.
